# Low-temperature aqueous-phase dehydrogenation of methanol catalyzed by synergistic Ir single-atom and cluster dual sites

**DOI:** 10.1093/nsr/nwaf585

**Published:** 2025-12-24

**Authors:** Xiaohui Liu, Xin Guan, Xiaolong Jia, Jingsen Bai, Wenjing Li, Xinying Li, Jianbing Zhu, Minhua Shao, Changpeng Liu, Meiling Xiao, Qing Jiang, Wei Xing

**Affiliations:** State Key Laboratory of Electroanalytic Chemistry, Jilin Province Key Laboratory of Low Carbon Chemistry Power, Jilin Provincial Science and Technology Innovation Center of Hydrogen Energy, Changchun Institute of Applied Chemistry, Chinese Academy of Sciences, Changchun 130022, China; School of Applied Chemistry and Engineering, University of Science and Technology of China, Hefei 230026, China; State Key Laboratory of Electroanalytic Chemistry, Jilin Province Key Laboratory of Low Carbon Chemistry Power, Jilin Provincial Science and Technology Innovation Center of Hydrogen Energy, Changchun Institute of Applied Chemistry, Chinese Academy of Sciences, Changchun 130022, China; State Key Laboratory of Electroanalytic Chemistry, Jilin Province Key Laboratory of Low Carbon Chemistry Power, Jilin Provincial Science and Technology Innovation Center of Hydrogen Energy, Changchun Institute of Applied Chemistry, Chinese Academy of Sciences, Changchun 130022, China; School of Applied Chemistry and Engineering, University of Science and Technology of China, Hefei 230026, China; State Key Laboratory of Electroanalytic Chemistry, Jilin Province Key Laboratory of Low Carbon Chemistry Power, Jilin Provincial Science and Technology Innovation Center of Hydrogen Energy, Changchun Institute of Applied Chemistry, Chinese Academy of Sciences, Changchun 130022, China; School of Applied Chemistry and Engineering, University of Science and Technology of China, Hefei 230026, China; State Key Laboratory of Electroanalytic Chemistry, Jilin Province Key Laboratory of Low Carbon Chemistry Power, Jilin Provincial Science and Technology Innovation Center of Hydrogen Energy, Changchun Institute of Applied Chemistry, Chinese Academy of Sciences, Changchun 130022, China; School of Chemistry and Life Science, Changchun University of Technology, Changchun 130022, China; State Key Laboratory of Electroanalytic Chemistry, Jilin Province Key Laboratory of Low Carbon Chemistry Power, Jilin Provincial Science and Technology Innovation Center of Hydrogen Energy, Changchun Institute of Applied Chemistry, Chinese Academy of Sciences, Changchun 130022, China; School of Applied Chemistry and Engineering, University of Science and Technology of China, Hefei 230026, China; State Key Laboratory of Electroanalytic Chemistry, Jilin Province Key Laboratory of Low Carbon Chemistry Power, Jilin Provincial Science and Technology Innovation Center of Hydrogen Energy, Changchun Institute of Applied Chemistry, Chinese Academy of Sciences, Changchun 130022, China; School of Applied Chemistry and Engineering, University of Science and Technology of China, Hefei 230026, China; CIAC-HKUST Joint Laboratory for Hydrogen Energy, Changchun Institute of Applied Chemistry, Chinese Academy of Sciences, Changchun 130022, China; Department of Chemical and Biological Engineering, The Hong Kong University of Science and Technology, Hong Kong 999077, China; CIAC-HKUST Joint Laboratory for Hydrogen Energy, Energy Institute, The Hong Kong University of Science and Technology, Hong Kong 999077, China; Guangzhou Key Laboratory of Electrochemical Energy Storage Technologies, Fok Ying Tung Research Institute, The Hong Kong University of Science and Technology, Guangzhou 511458, China; State Key Laboratory of Electroanalytic Chemistry, Jilin Province Key Laboratory of Low Carbon Chemistry Power, Jilin Provincial Science and Technology Innovation Center of Hydrogen Energy, Changchun Institute of Applied Chemistry, Chinese Academy of Sciences, Changchun 130022, China; School of Applied Chemistry and Engineering, University of Science and Technology of China, Hefei 230026, China; CIAC-HKUST Joint Laboratory for Hydrogen Energy, Changchun Institute of Applied Chemistry, Chinese Academy of Sciences, Changchun 130022, China; State Key Laboratory of Electroanalytic Chemistry, Jilin Province Key Laboratory of Low Carbon Chemistry Power, Jilin Provincial Science and Technology Innovation Center of Hydrogen Energy, Changchun Institute of Applied Chemistry, Chinese Academy of Sciences, Changchun 130022, China; School of Applied Chemistry and Engineering, University of Science and Technology of China, Hefei 230026, China; CIAC-HKUST Joint Laboratory for Hydrogen Energy, Changchun Institute of Applied Chemistry, Chinese Academy of Sciences, Changchun 130022, China; Key Laboratory of Automobile Materials, Ministry of Education, and School of Materials Science and Engineering, Jilin University, Changchun 130022, China; State Key Laboratory of Electroanalytic Chemistry, Jilin Province Key Laboratory of Low Carbon Chemistry Power, Jilin Provincial Science and Technology Innovation Center of Hydrogen Energy, Changchun Institute of Applied Chemistry, Chinese Academy of Sciences, Changchun 130022, China; School of Applied Chemistry and Engineering, University of Science and Technology of China, Hefei 230026, China; CIAC-HKUST Joint Laboratory for Hydrogen Energy, Changchun Institute of Applied Chemistry, Chinese Academy of Sciences, Changchun 130022, China

**Keywords:** low-temperature methanol reforming, liquid-phase reforming, heterogeneous catalysis, synergistic catalysis

## Abstract

Aqueous-phase reforming of methanol (APRM) offers a promising route for efficient hydrogen generation and safe transportation, yet it typically requires harsh conditions (above 200°C, 25–50 bar) and energy-intensive purification. Here, we report a heterogeneous catalyst featuring synergistic Ir single-atom and cluster dual sites that enables efficient hydrogen production from methanol and water at record-low temperatures (75°C–95°C) and ambient pressure. This unique ensemble effect drives a tandem reaction pathway, with Ir clusters promoting methanol dehydrogenation to formic acid, while adjacent Ir single atoms facilitate rapid formic acid decomposition into H_2_ and CO_2_ to suppress CO intermediates. As a result, the developed catalyst achieves a remarkable hydrogen production rate of 346.9 mol_H2_ mol_Ir_^−1^ h^−1^ and 100% H_2_ selectivity with no detectable CO formation. To the best of our knowledge, this represents one of the lowest temperature ranges demonstrated for efficient methanol-to-hydrogen conversion via heterogeneous catalysis, advancing methanol as a practical liquid H_2_ carrier for on-demand high-purity hydrogen production.

## Teaser

This work presents a dual-site iridium catalyst for low-temperature methanol dehydrogenation with nearly 100% H2 selectivity, opening an avenue to practical on-demand supply of hydrogen for clean energy devices.

## INTRODUCTION

Hydrogen (H_2_) has emerged as a compelling alternative to fossil fuels, yet the development of economical and sustainable H_2_ generation/storage technologies remains a critical obstacle for its global deployment [1–5]. In this context, various liquid organic carriers have been proposed to store hydrogen in the dense liquid phase and release hydrogen on demand, which offers an appealing methodology with high capacity and safety. Owing to the low-cost, easy-to handle and relatively high gravimetric hydrogen content (12.6% through direct dehydrogenation or 18.8% when reformed with water), methanol has been recognized as the most promising candidate among the reported liquid hydrogen carriers [6–12]. However, traditional methanol reforming processes typically suffer from severe limitations, including harsh operating conditions (>250°C, 25–50 bar) and inevitably generated carbon monoxide (CO) as a byproduct, which irreversibly poisons the fuel cell catalysts [13–17]. While recent advances in heterogeneous catalyst design have reduced operating temperature to ∼190°C, it is still challenging to further decrease the reforming temperature and increase the catalytic selectivity [18–20].

In contrast to the heterogeneously catalyzed methanol reforming, homogeneous catalysis enables aqueous-phase reforming of methanol (APRM) at low temperatures (<100°C) [9,12,21,22]. Pioneered by the work of Beller and colleagues, numerous metal complexes with various metal centers (Ru, Mn, Fe, Ir) and ligands have been developed and demonstrated enhanced activity and selectivity for APRM [23–26]. Despite the significant progress

achieved over the past decades, practical applications remain hindered by inherent drawbacks of molecular catalysts, such as prohibitive ligand costs, difficulties in separating the reaction system, and insufficient long-term stability [27,28]. These fundamental constraints highlight the urgent need to develop heterogeneous catalytic systems that combine the precision of molecular catalysis with the practicality of solid catalysts.

Our long-standing interest in efficient low-temperature dehydrogenation systems inspires us to design heterogeneous catalysts with well-defined active sites for the low-temperature APRM process [29–31]. Given that the APRM involves multiple elemental steps, such as initial methanol activation, hydration of formaldehyde followed by dehydrogenation generating formic acid, and the last dehydrogenation of formic acid, we strategically proposed a synergistic catalyst with tandem sites. As a proof of concept, we engineered a dual-site architecture combining iridium single atoms (Ir SA) and atomic clusters (Ir AC) on a nitrogen-doped carbon matrix (Ir_SA+AC_/NC). The Ir AC sites preferentially activate methanol dehydrogenation to formaldehyde, while neighboring Ir SA sites promote formic acid decomposition, which is confirmed by *operando* spectroscopy analysis and theoretical calculations. This unique synergy endows the as-developed Ir_SA+AC_/NC catalyst with unprecedented performance in low-temperature APRM, exhibiting a high turnover frequency (TOF) of 346.9 mol_H2_ mol_Ir_^−1^ h^−1^ under ambient conditions while completely suppressing CO formation. This work achieves a breakthrough in low-temperature hydrogen production by demonstrating the first heterogeneous catalytic system for APRM below 100°C, thereby establishing methanol as a technically viable hydrogen carrier for practical implementation.

## RESULTS AND DISCUSSION

### Catalyst synthesis and morphology characterization

**Figure 1.**
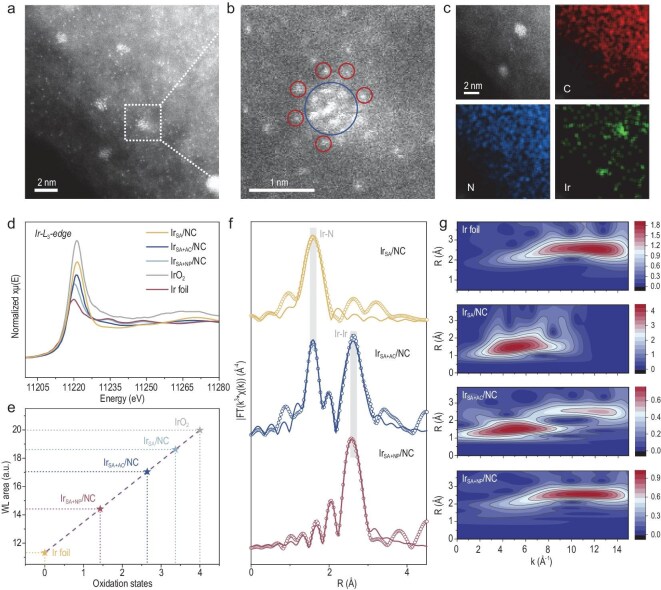
Structural characterization of the Ir/NC catalysts. (a and b) A high-resolution HAADF-STEM image (a) of Ir_SA+AC_/NC (enlarged in b) showing an iridium cluster (blue circle) and surrounding iridium atoms (red circles). (c) Energy-dispersive X-ray elemental mapping of Ir_SA+AC_/NC, indicating homogeneous distribution of iridium, nitrogen and carbon. (d) Normalized L_3_-edge XANES spectra of Ir_SA_/NC, Ir_SA+AC_/NC and Ir_SA+NP_/NC, with Ir foil and IrO_2_ as references. (e) Changes in the Ir valence state of Ir_SA_/NC, Ir_SA+AC_/NC and Ir_SA+NP_/NC, determined by comparison of the Ir L_3_-edge white line area with those of Ir foil and IrO_2_. (f) Fourier transforms of k^3^-weighted Ir L_3_-edge EXAFS spectra for Ir_SA_/NC, Ir_SA+AC_/NC and Ir_SA+NP_/NC. (g) Wavelet transforms of k^3^-weighted Ir L_3_-edge EXAFS signals for Ir foil, Ir_SA_/NC, Ir_SA+AC_/NC and Ir_SA+NP_/NC.

The as-proposed synergistic catalyst integrating both Ir single-atom Ir-N_4_ moieties (Ir SA) and atomic clusters (Ir AC) was engineered through a precisely controlled impregnation-pyrolysis strategy. Typically, zeolitic imidazolate framework-8 (ZIF-8) precursors were subjected to carbonization to yield the nitrogen-doped carbon host (NC), followed by another pyrolysis after controlled deposition of Ir species through wet-chemical impregnation. Scanning electron microscopy (SEM) confirms retention of the characteristic dodecahedral morphology with a uniform size of ∼200 nm, irrelative with the Ir feeding content (Fig. S1), while transmission electron microscopy (TEM) (Fig. S2) and X-ray diffraction (Fig. S3) analyses reveal an Ir dispersion transition from atomic to clustered and eventually nanoparticle (NP) states with increasing metal loading (Table S1). The resultant catalyst is denoted as Ir_SA_/NC, Ir_SA+AC_/NC and Ir_SA+NP_/NC, respectively. The coexistence of Ir SA and AC species at an intermediate Ir loading is unambiguously resolved by aberration-corrected high-angle annular dark-field scanning transmission electron microscopy (HAADF-STEM) (Fig. 1a). The enlarged image in Fig. 1b presents atomic-scale evidence of Ir single atoms (red circles) spatially organized around a central cluster (blue circle) with sub-nanometer proximity (<0.5 nm), creating an electronic transfer pathway between AC and SA sites. Statistical analysis reveals Ir clusters with an average size of 0.58 ± 0.13 nm (Fig. S4). The uniform dispersion of dual-active sites is further corroborated by energy-dispersive spectroscopy (EDS) elemental mapping, showing homogeneous Ir distribution across the nitrogen-doped carbon matrix (Fig. 1c and Fig. S5).

X-ray absorption fine structure spectroscopy (XAFS) analysis further reveals the structural evolution of Ir species in Ir/NC catalysts. Ir L_3_-edge X-ray absorption near-edge structure (XANES) spectra exhibit an absorption threshold shift toward metallic Ir foil with increasing Ir loading (Fig. 1d), indicating reduced Ir valence, consistent with the XPS results (Fig. 1e, Figs S6 and S7). Notably, the average Ir valence of Ir_SA+AC_/NC lies between metallic Ir foil and Ir_SA_/NC, further implying the successful construction of synergistic Ir SA and AC sites. Correspondingly, two scattering peaks at 1.58 (Ir–N) and 2.6 Å (Ir–Ir) are clearly discerned in the Fourier-transformed extended X-ray absorption fine structure (EXAFS) spectrum of the Ir_SA+AC_/NC catalyst (Fig. 1f, Figs S8 and S9). As Ir loading increases, Ir–Ir coordination number rises from 2.5 to 8.0, while Ir–N decreases from 4.2 to 1.9, signifying atomic Ir aggregation into clusters/nanoparticles. At an Ir loading of 4 wt%, the Ir–N and Ir–Ir coordination numbers are approximately 4.2 and 2.5, respectively (Table S2). In line with the EXAFS fitting results, wavelet transform (WT) analysis further confirms the coexistence of Ir–N and Ir–Ir coordination pathways within the Ir_SA+AC_/NC catalyst (Fig. 1g). Combined with TEM observations, these results demonstrate the coexistence of mononuclear Ir atoms and multinuclear Ir clusters stabilized on NC.

### Catalytic performance and stability

**Figure 2.**
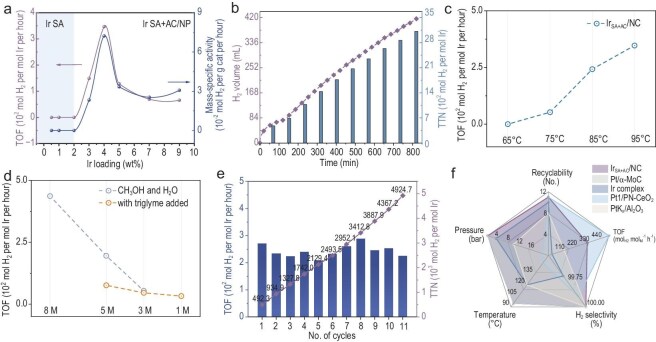
Catalytic performance for aqueous-phase methanol reforming. (a) Comparative catalytic activities of various Ir-based catalysts with and without atomically clustered Ir for hydrogen production from methanol. (b) Demonstration of a practical APRM process using 40 mg of Ir_SA+AC_/NC in a 4:1 MeOH/H_2_O solution containing 8.0 M KOH. TTN, total turnover number. (c) Methanol reforming activity of Ir_SA+AC_/NC at different reaction temperatures. (d) Methanol reforming activity of Ir_SA+AC_/NC under varying base concentrations, with and without additives. (e) Recyclability of the Ir_SA+AC_/NC catalyst in repeated cycles of hydrogen production from methanol. (f) Comparison of key factors in the APRM reaction (recyclability, TOF, H_2_ selectivity, temperature and pressure) for Ir_SA+AC_/NC, Pt/α-MoC [18], Ir complex [32], Pt_1_/PN-CeO_2_ [33] and PtK_x_/Al_2_O_3_ [34] catalysts.

The catalytic performance of a series of Ir/NC samples for APRM process (95°C, ambient pressure) was systematically evaluated, revealing a striking structure-activity dependency (Fig. 2a and Table S3). While pure NC and the Ir_SA_/NC catalyst remain inactive, the introduction of either Ir nanoclusters or nanoparticles drives efficient APRM conversion, highlighting the critical role of metallic Ir in facilitating the reaction. TOF follows a volcano-type trend with increasing Ir loading. The optimal Ir_SA+AC_/NC (4 wt% Ir) achieves a remarkable TOF of 346.9 h^−1^, representing more than a 3-fold enhancement over counterparts with 3 wt% (148.9 h^−1^) and 5 wt% (128.3 h^−1^) Ir loadings. The trend reflects an Ir-loading-dependent optimization of dispersion and cluster size, and a moderate loading (4 wt%) stabilizes a cooperative ensemble of atomically dispersed and ultrasmall clustered Ir species, creating a synergistic architecture that maximizes catalytic performance (Fig. S10 and Table S2).

Systematic optimization of the MeOH/H_2_O ratio and KOH concentration (Table S3) reveals optimal performance for the Ir_SA+AC_/NC catalyst in 8.0 M KOH at an MeOH/H_2_O ratio of 4:1 (Fig. 2b). Base addition accelerates the reaction while concurrently elevating the boiling point temperature (Fig. 2c, Table S4 and Figs S11 and S12); introducing a co-solvent moderates alkalinity and locks the system at a stable temperature, maintaining H_2_ production with undetectable CO levels and only a modest decline in rate (Fig. 2d). Crucially, all processes generate CO-free hydrogen (Fig. S13) with sufficient purity to directly power an H_2_/O_2_ fuel cell (Fig. S14), substantively improving both the environmental sustainability and the economic feasibility of the APRM process. To the best of our knowledge, this represents one of the lowest temperatures (95°C) reported for efficient heterogeneous methanol–water reforming under purely thermal conditions (Table S5).

To validate practical APRM implementation targeting a 3:1 H_2_/CO_2_ gas ratio, we conducted *operando* monitoring of the evolved gas. Initially, the majority of generated CO_2_ was captured in solution as carbonate species. Progressive pH decrease triggered CO_2_ release, yielding stable gas distribution and pH equilibrium. As anticipated, high-purity H_2_ was detected within 2 h, whereas CO_2_ was released after acidification with HCl (Figs S15–S17). The final H_2_/CO_2_ ratio reached 2.87:1, approaching the theoretical 3:1 value. Catalytic stability of Ir_SA+AC_/NC—a crucial factor for heterogeneous catalysts—was evaluated through a cycling test (Fig. 2e), during which H_2_ evolution decreases modestly from 270 to 225 mol_H2_ mol_Ir_^−1^ h^−1^, while H_2_ selectivity remains at 100%. Post-reaction analysis of the spent Ir_SA+AC_/NC indicates a moderate decline in TOF correlates with restructuring of Ir from single atoms to atomically dispersed clusters, rather than with extensive Ir dissolution (Figs S18–S21, Tables S6 and S7).

The Ir_SA+AC_/NC catalyst outperforms state-of-the-art low-temperature APRM catalyst systems, including Pt/α-MoC [18], Ir complex [32], Pt_1_/PN-CeO_2_ [33] and PtK_x_/Al_2_O_3_ [34] catalysts in multiparameter benchmarking (Fig. 2f). Across five key metrics (recyclability, TOF, H_2_ selectivity, temperature and pressure), the Ir_SA+AC_/NC catalyst demonstrates superior performance, achieving five times higher TOF than Ir complex under identical conditions, thus highlighting its exceptional efficiency in pure hydrogen production under mild conditions.

### *In situ* spectroscopic analysis

**Figure 3.**
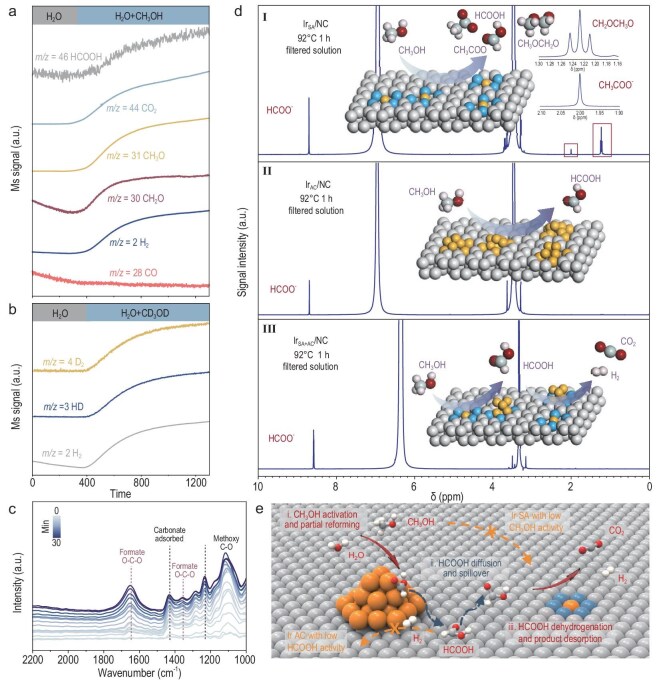
*In situ* NMR, ATR-SEIRAS and isotope-labeled mass spectrometry experiments over Ir/NC. (a) *In situ* mass spectrometry signals of H_2_ (*m*/*z* = 2), CO (*m*/*z* = 28), CH_2_O (*m*/*z* = 30), CH_3_O (*m*/*z* = 31), CO_2_ (*m*/*z* = 44) and HCOOH (*m*/*z* = 46) recorded during APRM over Ir_SA+AC_/NC at room temperature. (b) *In situ* mass spectrometry signals of H_2_ (*m*/*z* = 2), HD (*m*/*z* = 3) and D_2_ (*m*/*z* = 4) recorded after isotope labeling during APRM over Ir_SA+AC_/NC at room temperature. (c) Time-resolved *in situ* ATR-SEIRAS spectra of Ir_SA+AC_/NC collected during APRM at room temperature. (d) ^1^H NMR spectra (MeOH/H_2_O 4:1, KOH 8 M, CD_3_OD) of the reaction mixture over (I) Ir_SA_/NC, (II) Ir_AC_/NC and (III) Ir_SA+AC_/NC catalysts, recorded at room temperature. The values reported on the spectra refer to the reaction temperature at the time of sample withdrawal. The inset includes a schematic illustration of the possible dominant reaction on each catalyst. (e) Proposed mechanistic model for APRM, highlighting the synergistic effect of Ir AC and Ir SA sites.

*In situ* spectroscopy was employed to probe the activation of water and methanol over the Ir_SA+AC_/NC catalyst by monitoring surface adsorbate evolution. Isotope-labeled *in situ* mass spectrometry (Fig. 3a) shows that introducing methanol into the catalyst/water mixture at room temperature triggers a rapid increase in the ionic current at *m*/*z* = 2 (H_2_), accompanied by intensified signals at *m*/*z* = 31 (CH_3_O) and *m*/*z* = 30 (CH_2_O), confirming methanol dissociative adsorption and hydrogen production. The absence of signals at *m*/*z* = 29 (CHO) and *m*/*z* = 28 (CO) suggests suppression of CH_2_O decomposition, a critical step enabling formic acid formation. Concurrently, signals at *m*/*z* = 44 (CO_2_) and *m*/*z* = 46 (HCOOH) confirm CH_2_O reforming with H_2_O to formic acid and its subsequent dehydrogenation to CO_2_ and H_2_. When CD_3_OD and H_2_O were co-fed, the simultaneous emergence of H_2_, HD and D_2_ (Fig. 3b) unequivocally evidences the cleavage of both C–H and O–H bonds and water-assisted complete dehydrogenation pathway. Furthermore, complementary ^13^C and ^18^O labeling studies confirm that the carbon in the products originates solely from methanol, while the oxygen is derived from both methanol and water (Fig. S22).

Further mechanistic insight was gained through *in situ* attenuated total reflectance-surface-enhanced infrared absorption spectroscopy (ATR-SEIRAS). As shown in Fig. 3c, characteristic bands appear at 1000–1200 cm^−1^ upon methanol introduction, which are assigned to the C–O stretching vibration (νC–O) of methoxy species [35,36]. Concurrently, distinct peaks at 1355 and 1641 cm^−1^ corresponding to O–C–O symmetric [ν_s_(OCO)] and asymmetric [ν_as_(OCO)] stretching vibrations of formate species [37,38], and bands at 1232 and 1434 cm^−1^ assigned to carbonate species [39,40] are discerned. As the reaction proceeds, formate-related peaks (1355 and 1641 cm^−1^) intensified significantly, while no CO intermediates are detected (2000–2100 cm^−1^). This indicates that formate species serve as a pivotal intermediate that effectively circumvents CO formation and ensures complete H_2_ selectivity. The concurrent increase in carbonate band intensity further verifies room-temperature conversion of formic acid to CO_2_ and H_2_, consistent with its exceptional low-temperature catalytic activity in APRM.

Complementary *in situ* nuclear magnetic resonance (NMR) studies were conducted to examine the synergistic effect of dual sites in APRM (Fig. 3d). We used Ir_SA_/NC and Ir_AC_/NC catalysts, each containing isolated Ir SA and Ir AC sites, respectively (Figs S23–S26). For the Ir_SA_/NC catalyst, peaks corresponding to formate (8.5–9.0 ppm), CH_3_OCH_2_O (1.18–1.28 ppm) and acetate (CH_3_COO^−^, 2.00 ppm) were clearly identified once the catalyst was introduced into the methanol/water mixture, indicating competitive C–C coupling that impeded methanol reforming. Conversely, only formate-related peaks, without other byproducts, were detected in the spectrum of Ir_AC_/NC, implying that the negligible APRM activity of Ir_AC_/NC arises from its inability to dehydrogenate formic acid, as validated by correlative analysis of formic acid dehydrogenation (Figs S27–S29). Critically, only the dual-site Ir_SA+AC_/NC catalyst achieves complete methanol reforming, leveraging a synergistic mechanism (Fig. 3e), wherein Ir AC efficiently activate methanol to formic acid initially and adjacent Ir SA subsequently dehydrogenate formic acid to H_2_ and CO_2_. This cooperative interplay between atomic and clustered sites is essential for efficient low-temperature methanol reforming.

### Density functional theory (DFT) mechanistic insights

**Figure 4.**
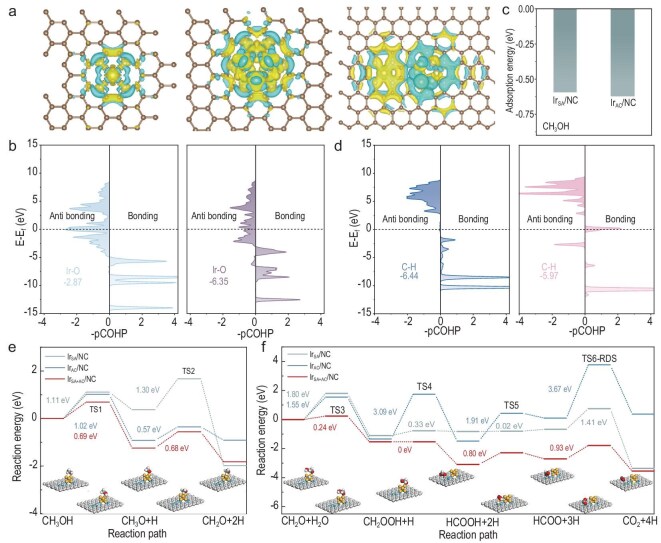
First-principles calculations of Ir/NC catalysts. (a) Charge density distributions of occupied d-bands for Ir_SA_/NC, Ir_AC_/NC and Ir_SA+AC_/NC, ranging from −1 eV to the Fermi level (set as 0). (b) −COHP of the Ir–O bond for CH_3_O* adsorption on Ir_SA_/NC (left) and Ir_AC_/NC (right). (c) Adsorption energies of CH_3_OH on Ir_SA_/NC and Ir_AC_/NC. (d) −COHP of the C–H bond for HCOO* adsorption on Ir_AC_/NC (left) and Ir_SA_/NC (right). (e) Energy profiles for CH_3_OH dissociation into CH_2_O and H atoms on Ir_SA_/NC, Ir_AC_/NC and Ir_SA+AC_/NC surfaces. (f) Energy profiles for CO_2_ formation via the CH_2_O and H_2_O reaction on these three surfaces. The *x*-axis shows the reaction intermediates and transition states (TS); the *y*-axis shows the relative energy of each state. Ir, N, C, O and H atoms are shown in orange, blue, gray, red and white, respectively.

Periodic DFT calculations were further performed to elucidate the underlying catalytic mechanisms. A graphene-supported model featuring an Ir–N_4_ site adjacent to an Ir_6_–N_X_ site was constructed to mimic Ir_SA+AC_/NC, while isolated Ir–N_4_ and Ir_6_–N_X_ were built to represent Ir_SA_/NC and Ir_AC_/NC, respectively (Fig. 4a). The calculated average Mulliken charges were +0.89 |e| for Ir_SA_/NC, +0.30 |e| for Ir_AC_/NC and +0.94 |e| and +0.35 |e| for the mononuclear and cluster sites in Ir_SA+AC_/NC, respectively. Charge density analysis further indicates that occupied d-band states near the Fermi level are predominantly localized on low-coordination Ir atoms, where activation of O–H bonds preferentially occurs.

The Ir–O bonding characteristics during CH_3_O adsorption were analyzed using crystal orbital Hamiltonian population (COHP), where negative values (−COHP) represent bonding states (Fig. 4b). The integrated COHP values (ICOHP), a descriptor of Ir–O bond strength, were determined to be −2.87 and −6.35 eV for Ir_SA_/NC and Ir_AC_/NC, respectively. Similar trends were observed for CH_3_OH adsorption, which is stronger on Ir_AC_/NC than on Ir_SA_/NC (Fig. 4c), suggesting higher CH_3_OH dehydrogenation activity at Ir AC sites. By contrast, linearly adsorbed formate on isolated Ir SA sites exhibits a less-negative integrated COHP than on Ir AC sites (ICOHP −6.44 → −5.97 eV; Fig. 4d) and a calculated C–H-cleavage barrier lower by ca. 2.3 eV, thereby accounting for the superior dehydrogenation activity of the Ir SA sites. Notably, the synergistic Ir_SA+AC_/NC model reproduces these trends: for methoxy adsorption, ICOHP(Ir–O) is more negative at Ir AC than at Ir SA (−4.22 vs. −1.57 eV), whereas along the formate dehydrogenation coordinate ICOHP(C–H) is less negative at Ir SA than at Ir AC (−5.44 vs. −6.43 eV), indicating a weaker C–H bond and more facile cleavage at Ir SA (Fig. S30) and thereby supporting an SA–AC cooperative mechanism.

Detailed reaction pathway calculations elucidate the mechanistic roles of Ir SA and Ir AC sites in methanol dehydrogenation. Significant kinetic barriers occur on Ir SA during *CH_3_O dehydrogenation and CH_2_OOH formation, whereas Ir AC sites lowers the *CH_3_O dehydrogenation barrier by 0.62 eV, facilitating the initial activation (Fig. 4e and Fig. S31). Following *CH_2_O generation, the reaction proceeds via two competing pathways: (i) producing *CH_2_OOH and *H; and (ii) reaction with excess methanol to yield CH_3_OCH_2_O and CH_3_COO^−^. Low-temperature CH_2_O–H_2_O reforming over Ir SA is kinetically hindered (*E*_a_ = 1.80 eV), diverting the reaction toward C–C coupling and hence C_2_ formation, in agreement with experimental results. Conversely, Ir_6_–N_X_ reduces the CH_2_OOH formation barrier to below 0.24 eV, though further dehydrogenation is kinetically hindered under moderate conditions due to a high C–H cleavage barrier (3.09 eV). In the synergistic Ir_SA+AC_/NC catalyst, Ir AC promotes methanol dissociation and formic acid formation, while Ir SA catalyzes the subsequent formic acid dehydrogenation. Weakly adsorbed CH_2_OOH migrates from Ir AC to adjacent Ir SA sites, where pathway convergence enables rapid *CH_2_OOH decomposition without thermodynamic barrier. The rate-determining step is identified as the cleavage of the final C–H bond in formic acid, with an associated barrier of 0.93 eV, which is consistent with experimental observations (Fig. 4f).

## CONCLUSION

In summary, we develop a dual-site Ir_SA+AC_/NC catalyst integrating atomically dispersed Ir sites and sub-nanometric Ir clusters for efficient APRM under mild conditions (95°C and 1 bar). This architecture achieves unprecedented activity with a TOF of 346.9 h^−1^ while completely suppressing CO. *In situ* spectroscopy and DFT calculations reveal a tandem mechanism enabled by the synergetic sites: Ir AC sites dehydrogenate methanol to HCOOH with significantly decreased energy barrier, while adjacent Ir SA sites facilitate rapid HCOOH decomposition. By coupling single-atom and clustered sites electronically and geometrically, this work establishes a heterogeneous platform for low-temperature methanol dehydrogenation chemistry and pioneers an on-demand hydrogen delivery strategy from liquid hydrogen carriers.

## Supplementary Material

nwaf585_Supplemental_File
