# Supplementary material for: Low-temperature aqueous-phase dehydrogenation of methanol catalyzed by synergistic Ir single-atom and cluster dual sites
Source: Natl Sci Rev. 2025 Dec 24;13(4):nwaf585. doi: 10.1093/nsr/nwaf585 (PMC12902689; doi:10.1093/nsr/nwaf585)
Supplement: nwaf585_Supplemental_File [file nwaf585_supplemental_file.pdf]

Supplemental information

Low-temperature aqueous-phase dehydrogenation of methanol catalyzed by synergistic Ir single-atom and cluster dual sites

1. Supplementary Methods

1.1 Synthesis of NC.

ZIF-8 were prepared by rapidly adding 40 mL of a methanol solution containing 3 g of $Zn(NO_3)_2$ $6H_2O$ to 80 mL of a 2-methylimidazole solution (6.5 g) while stirring. The mixture was stirred for 24 h. The resulting white precipitate was centrifuged, washed three times with ethanol, and dried under vacuum at 60 ℃. The resulting ZIF-8 powder was ground and subjected to heat treatment at 950 ℃ for 1 h under a flow of Ar/H_2 (10%) gas in a tube furnace. The obtained brown powder was then dispersed in 100 mL of DI water, and 10 mL of concentrated hydrochloric acid (36–38%) was added under ultrasonic vibration. The mixture was stirred at 80 ℃ for an additional 12 h, followed by washing and vacuum filtration. The NC powder was obtained after drying overnight at 60 ℃.

1.2 Synthesis of Ir AC.

The precursor for the Ir atomic clusters (AC) was prepared via a wet-chemistry hydrothermal process. Specifically, 100 mg of PVP was dispersed in 15 mL of ethylene glycol, and then 660 μL (0.06 mmol) of H_2IrCl_6 solution (17.5 mg Ir mL^{-1}) was added dropwise. After stirring for 30 min, the mixture was transferred to a 25 mL Teflon-lined stainless steel autoclave. The autoclave was sealed and heated at 200 ℃ for 12 h. Finally, the Ir AC were centrifuged, washed several times with DI water and ethanol, and dried overnight in air at 60 ℃.

1.3 Synthesis of Ir_{SA}/NC, Ir_{SA+AC}/NC, Ir_{SA+NP}/NC, and Ir_{AC}/NC.

50 mg of NC was added to 30 mL of a 1:1 isopropanol and DI water solution and dispersed by ultrasound to obtain a black solution. Then, a solution of H_2IrCl_6 (17.5 mg Ir mL^{-1}) corresponding to the desired mass fraction was added dropwise to the black solution under stirring for 10 h. The product was dried at 120 ℃ to remove the solvent. The resulting black powder was then mixed with 250 mg of melamine as an additional nitrogen source, ground, and heated at 900 ℃ for 1 hour in an Ar/H_2 (10%) gas mixture to yield Ir_{SA}/NC, Ir_{SA+AC}/NC, and Ir_{SA+NP}/NC. Ir_{AC}/NC was prepared by a similar procedure as Ir_{SA+AC}/NC, except that the H_2IrCl_6 solution was replaced with Ir AC.

Mechanistically, the ZIF-8 derived NC provides N_4-type coordination cavities (created as Zn volatilizes) that anchor Ir(III) during activation. Under low Ir loading, Ir(III) from a dilute precursor preferentially binds these N_4 sites, yielding isolated Ir-N_4 centers (Ir_{SA}/NC). As the Ir concentration in the precursor increases and the Ir/N_4 ratio approaches or exceeds the anchoring capacity, excess Ir lacking high-affinity sites-nucleates into atomic clusters adjacent to single atoms (Ir_{SA+AC}/NC) and, at still higher loadings, coarsens into nanoparticles (Ir_{SA+NP}/NC). Throughout, melamine serves as a transient nitrogen source that replenishes surface N, strengthens Ir-N coordination, and suppresses Ir migration during the thermal step, thereby promoting high single-atom dispersion.

2. Characterization

Structural characterizations. Scanning electron microscopy (SEM) measurements were performed with an XL 30 ESEMFEG field emission scanning electron microscope. Transmission electron microscopy (TEM), high-resolution transmission electron microscopy (HRTEM), and elemental mapping analysis were performed on a Thermo Scientific talos f200s electron microscope operating at 200kV. High-annular dark-field scanning transmission electron microscopy (HAADF-STEM) images were performed on a Titan 80-300 scanning/transmission electron microscopy operated at 300 kV. The surface electronic states were determined by X-ray photoelectron spectroscopy (XPS) using a Thermo Scientific ESCALAB 250Xi using Al Kα irradiation. XRD patterns were recorded on Rigaku miniflex 600 with Cu Kα irradiation. Inductively coupled plasma optical emission spectroscopy (ICP-OES; X Series 2, Thermo Scientific USA) was used to determine the ratio of Ir in the as-prepared samples.

XAFS data collection and analysis. X-ray absorption fine structure (XAFS) spectra was recorded on the beam line at the Shanghai Synchrotron Radiation Facility (SSRF), Shanghai Institute of Applied Physics, China. The Ir L_3-edge spectra of the samples were collected in transmission mode using a Lytle detector. The XAFS raw data were background-subtracted and normalized by the ATHENA program. Least-squares curve-fitting analysis of the EXAFS χ(k) data (including different coordination shells, in the R-space (1.0 - 3.1 Å) and Fourier transforms (in the k-space 2.8 - 13.0 $Å^{-1}$) were carried out using the ARTEMIS program.

3. Catalyst Testing

All reactions were carried out under an inert atmosphere (nitrogen) to exclude air. The reaction mixture was heated to 95 ℃ at atmospheric pressure, and once the system stabilized, the catalyst was added. The gas production was continuously monitored in real time using a micro gas flow meter (calibrated prior to the experiment). Simultaneously, the gas phase was analyzed using a Thermo Scientific Trace 1600 gas chromatograph equipped with a methanizer-flame ionization detector (FID) and a thermal conductivity detector (TCD). After the experiment, the reaction mixture was filtered and then analyzed.

The turnover number (TON) was calculated based on the amount of hydrogen gas produced, where each hydrogen molecule represents one turnover. Specifically, the TON was calculated using the following equation:

$$TON = \frac{V_{\mathrm{obsved}} - V_{\mathrm{blank}}}{V_{\mathrm{m},H_2,25℃} * \mathrm{nIr}}$$

V_{obsved}: measured gas volume from micro gas flow meter (mL)

V_{blank}: the blank colume (mL)

nIr： amount of Ir in catalyst （mmol）

$V_{\mathrm{m,H2,25℃}}$:molar gas volume, $V_{\mathrm{m,H2,25℃}}$=24.49mL/mmol at 25℃ calculated from the van der waals

equation:

$$V_{m,H_2,25℃} = \frac{RT}{p} + b - \frac{a}{RT} = 24.49\frac{ml}{mmol}$$

For hydrogen gas, the parameters are R= 8.3145 m^3 Pa mol^{-1} K^{-1}, T= 298.15 K, p= 101325 Pa, a= 2.49 $\cdot 10^{-10}$ Pa m^3 mol^{-2}, b= 26.7 $\cdot 10^{-6}$ m^3 mol^{-1}.

4. Product and reaction intermediates Analysis

^{1}H NMR spectra were obtained at 300 MHz on a Bruker AVANCE 300 spectrometer, with CD_3OD added for locking and shimming purposes. Gaseous products were analyzed using a Thermo Scientific Trace 1600, equipped with a methanizer unit and both FID and TCD detectors. To obtain an accurate gas ratio, the gaseous products were diluted with high-purity N_2 gas.
To detect potential reaction intermediates, the reaction was performed at different temperatures. Ir_{SA}/NC, 4% Ir_{SA+AC}/NC and Ir_{AC}/NC were separately placed in a two-necked flask equipped with a reflux condenser. A 5 mL mixture of MeOH/H_2O (4:1) and an 8 M KOH solution were added, and the resulting mixture was stirred at room temperature for 1h. A 1 mL aliquot of the reaction mixture was taken using a syringe fitted with a filter and transferred to an NMR tube, to which CD_3OD was added. The remaining liquid was heated, and when the temperature reached 80 ℃, a sample was withdrawn and prepared for NMR analysis using the same procedure. At 92 ℃, a large amount of gas was observed to evolve. After 30 minutes at 92 ℃, a sample was taken for NMR analysis.

***In situ* mass measurements** were performed using a system provided by Linglu Instruments (Shanghai) Co. Ltd to detect intermediates and products during the APRM process. This system ensures fast transport of species formed at the catalyst surface to the mass spectrometric compartment, where volatile products are evaporated into the vacuum system of the mass spectrometer with a delay time of 1 second. In a typical test, 1 mL of deionized water and 2 mg of catalyst are added to the test cell, and the signal is allowed to stabilize. Then, 0.5 mL of MeOH is added to the test cell, and signal fluctuations in the mass spectrometer are recorded. Isotope labeling experiments are conducted in the same manner, but the corresponding solution is replaced.

***In situ* ATR-SEIRAS** was conducted with time-resolved FT-IR spectrometer (Nicolet iS50; Thermo Scientific USA) equipped with a liquid nitrogen-cooled Mercury Cadmium Telluride (MCT) detector. A methanol–water mixed solution saturated with N_2, which was continuously purged with N_2 during the test. IR spectra were collected using unpolarized IR radiation with a spectral resolution of 4 cm^{-1} and a collection time of 30 seconds.

5. Computation Details

To detect potential reaction intermediates, the geometries and energies were calculated by density functional theory (DFT) [1] implemented in the CASTEP code with ultrasoft pseudopotentials [2].

The exchange-correlation functional was the Perdew-Burke-Ernzerhof (PBE) [3] functional combined with the TS method [4]. The cutoff energy was set to 400 eV and the Brillouin zone was sampled by 1x1x1 k-points with the Monkhorst-Pack scheme. The convergence threshold was set to 1E-5 eV atom^{-1} for energy and the max force was set to 0.05 eV Å^{-1}. Spin polarization was considered in the study. We performed the linear and quadratic synchronous transit (LST/QST) algorithms with subsequent conjugate gradient methods to study the transition state [5].
The metal-particle (Ir_6) was constructed by cutting the bulk metal (Ir) in a sphere with a radius of 2.5 Å. The Ir_6 cluster was inserted into the defect site of graphene (delete two C atoms) near the IrN_4, and a vacuum layer of 15 Å was used along the z direction to avoid periodic interactions.

6. Supplementary Figures and Tables

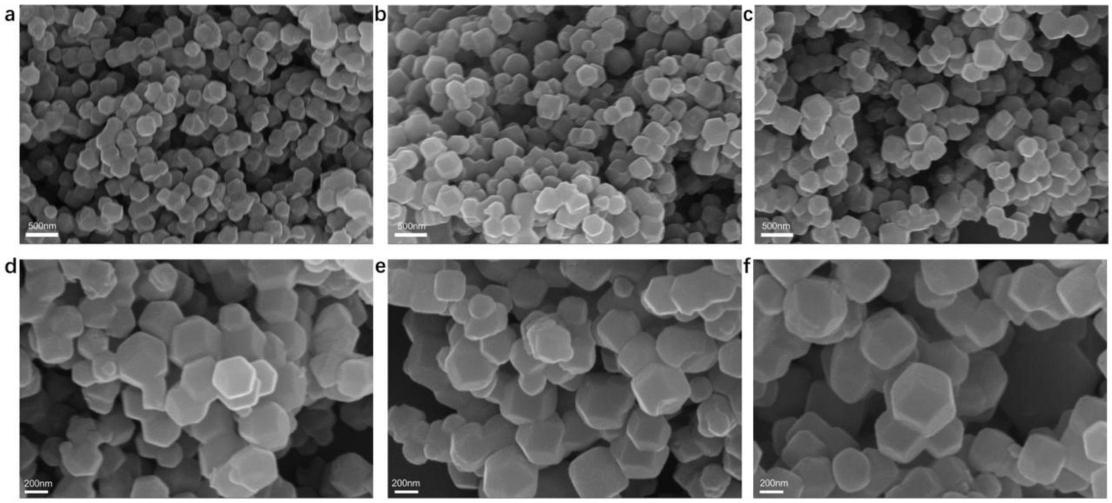

Figure S1. Morphological characterization of the catalysts. SEM images of (a, d) Ir_{SA}/NC, (b, e) Ir_{SA+AC}/NC, and (c, f) Ir_{SA+NP}/NC.

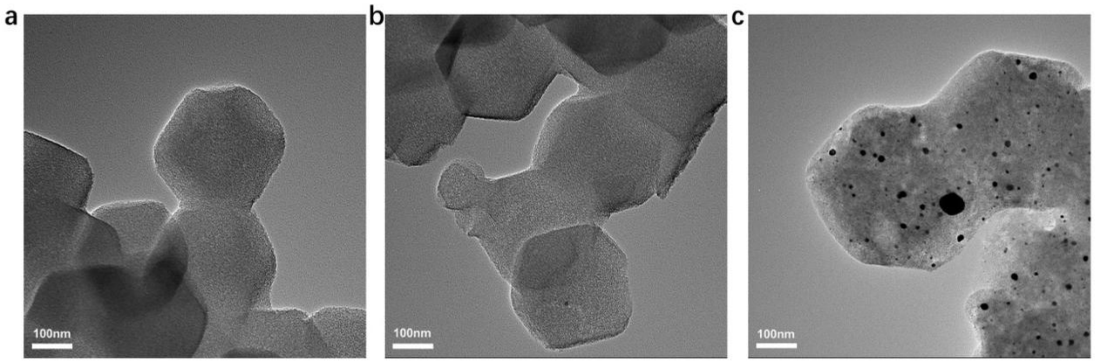

Figure S2. Morphological characterization of the catalysts. TEM images of (a) Ir_{SA}/NC, (b) Ir_{SA+AC}/NC, and (c) Ir_{SA+NP}/NC.

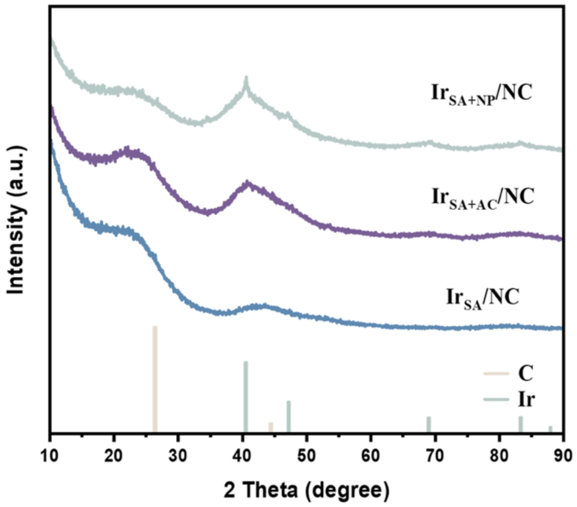

Figure S3. X-ray diffraction (XRD) patterns of Ir$_{SA}$/NC, Ir$_{SA+AC}$/NC, and Ir$_{SA+NP}$/NC.

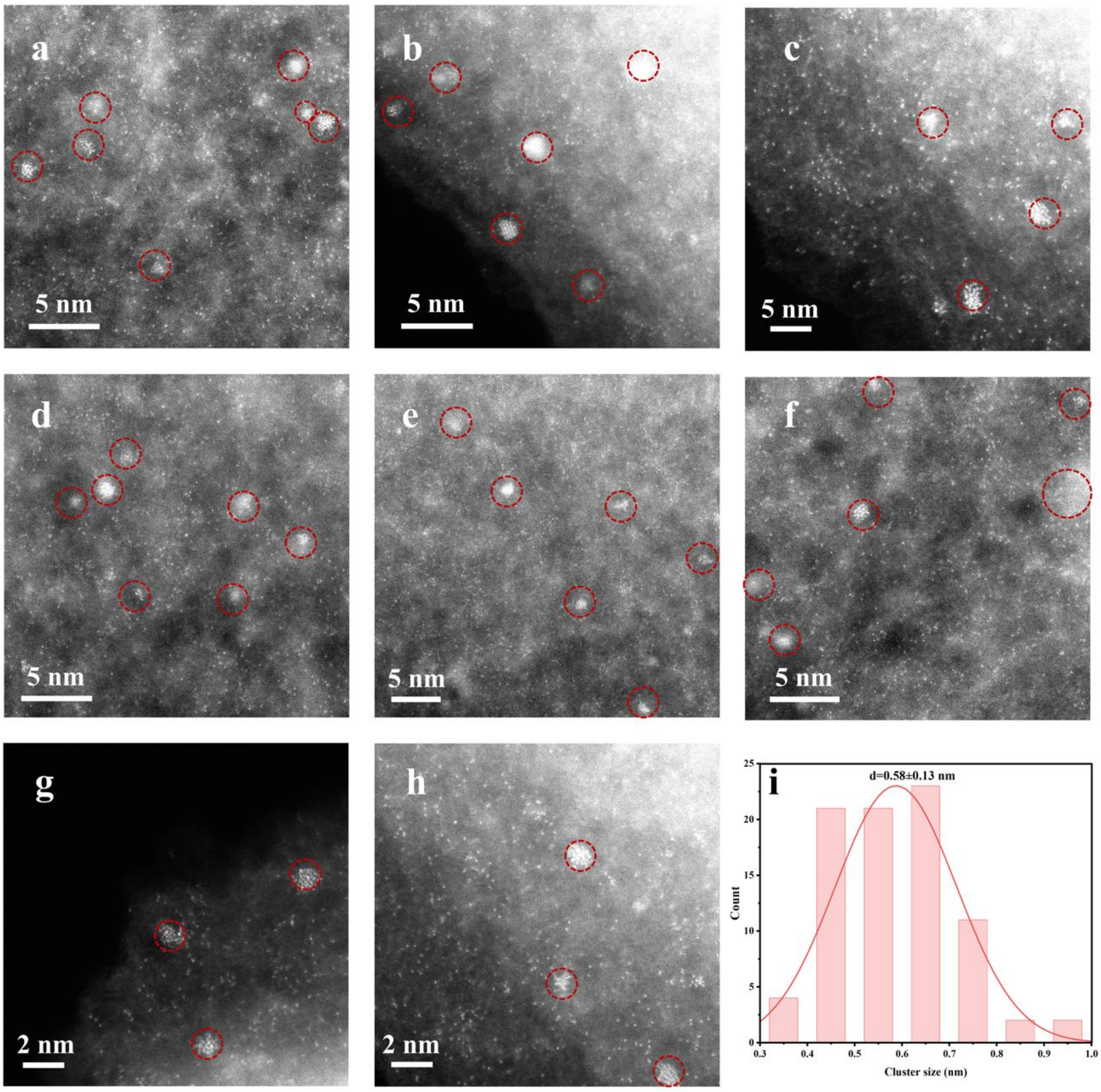

Figure S4. Cluster size statistics. (a-h) Different areas of HAADF-STEM images used for the statistics. (i) The average cluster size of Ir on Ir_{SA+AC}/NC is estimated to be 0.58 ±0.13 nm.

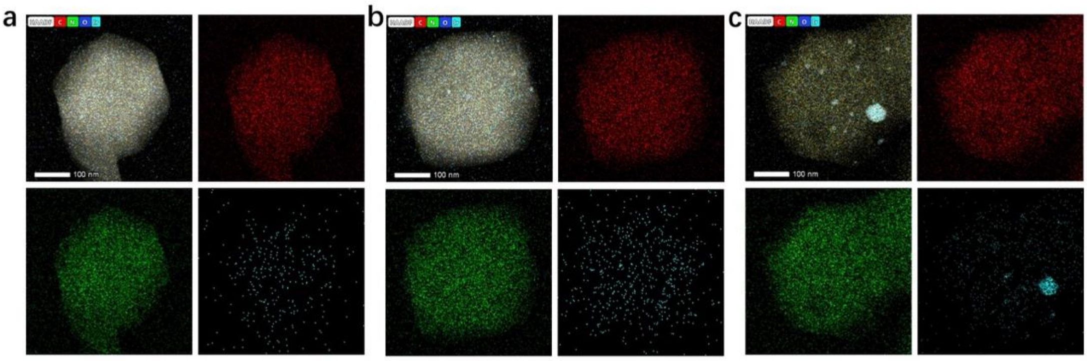

Figure S5. EDS element mapping. Elemental mapping of Ir, N, and C in a randomly chosen (a) Ir_{SA}/NC, (b) Ir_{SA+AC}/NC, and (c) Ir_{SA+NP}/NC, showing the uniform dispersion of Ir on the surface of NC. As the Ir content increases, distinct nanoparticles are formed.

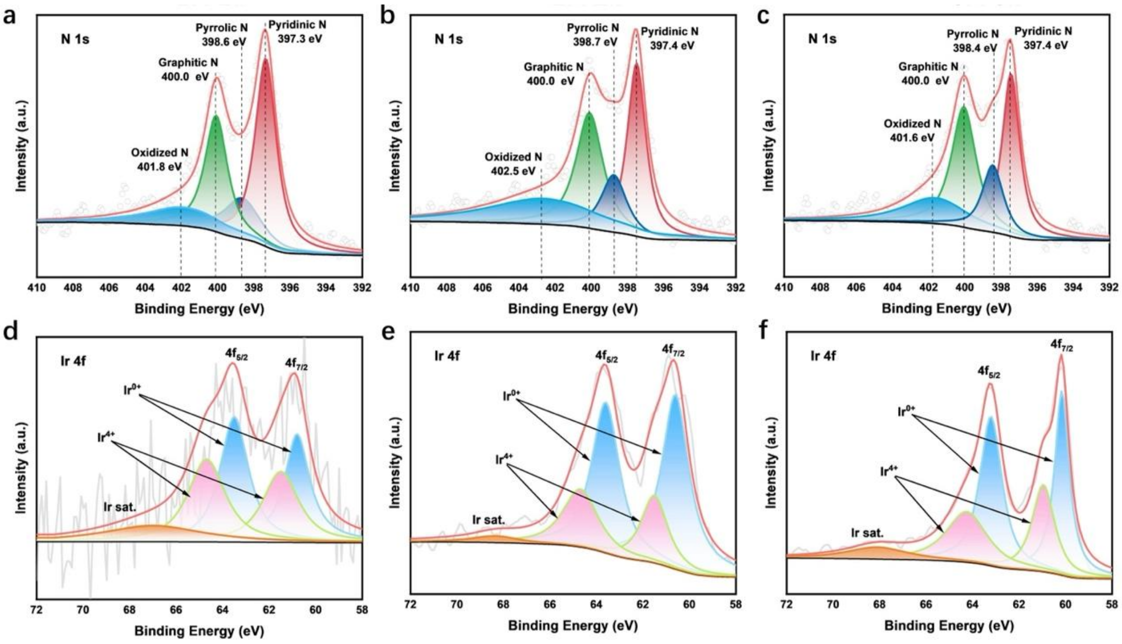

Figure S6. N 1s XPS spectra for (a) Ir_{SA}/NC, (b) Ir_{SA+AC}/NC, and (c) Ir_{SA+NP}/NC; Ir 4f XPS spectra for (d) Ir_{SA}/NC, (e) Ir_{SA+AC}/NC, and (f) Ir_{SA+NP}/NC.

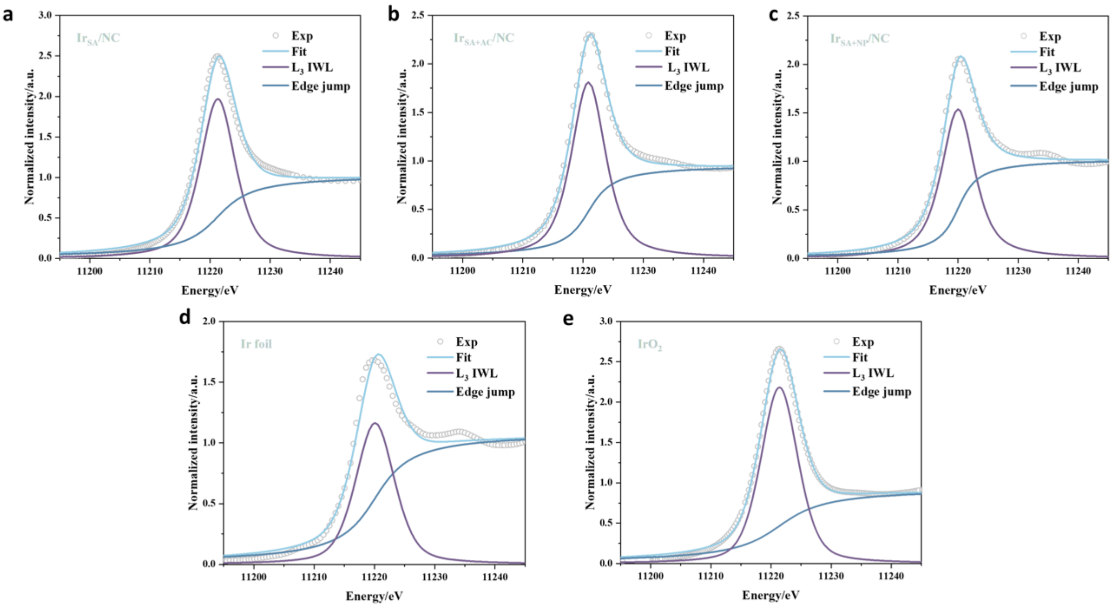

Figure S7. The Ir L_3-edge XAS white line peak integration results for Ir_{SA}/NC, Ir_{SA+AC}/NC, Ir_{SA+NP}/NC, Ir foil, and IrO_2 samples.

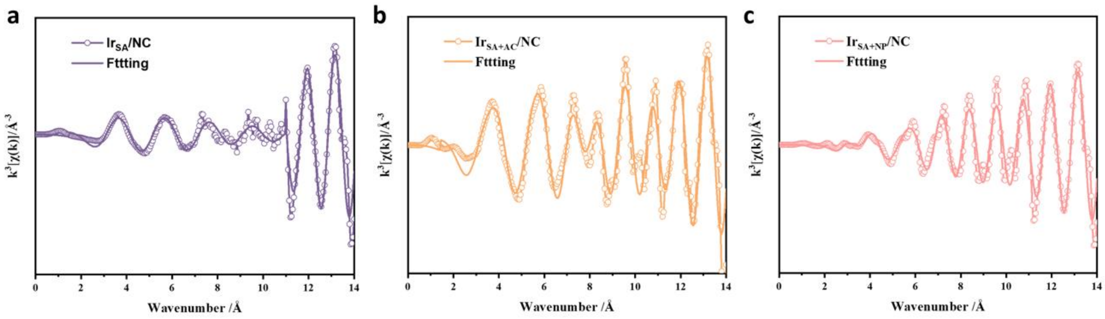

Figure S8. K-space EXAFS spectra. (a) EXAFS fitting of IrSA/NC in k-space. (b) EXAFS fitting of Ir_{SA+AC}/NC in k-space. (c) EXAFS fitting of Ir_{SA+NP}/NC in k-space.

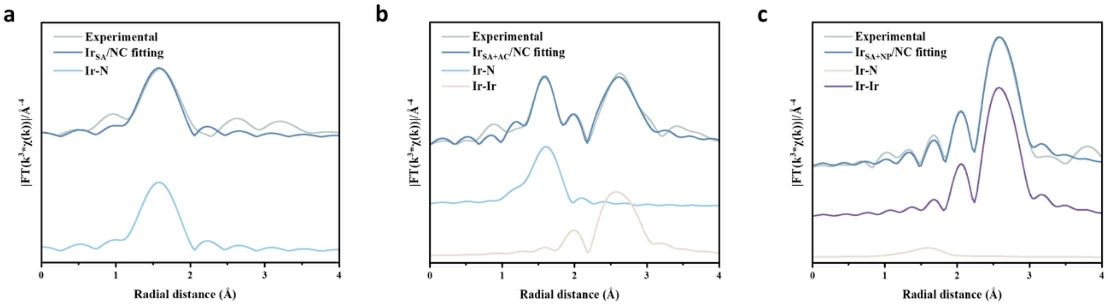

Figure S9. EXAFS fitting results of (a) Ir_{SA}/NC, (b) Ir_{SA+AC}/NC and (c) Ir_{SA+NP}/NC at the Ir L_3-edge, with the scattering path presented.

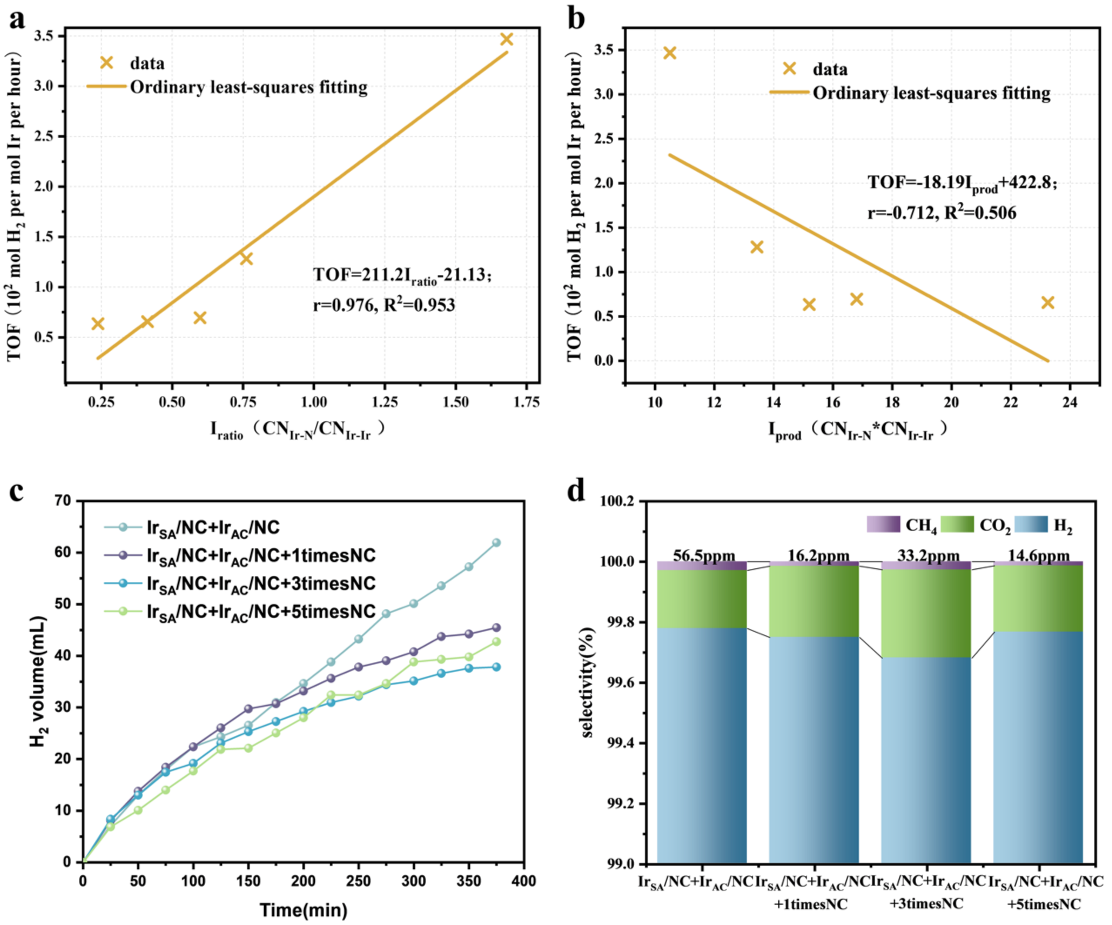

Figure S10. (a) Correlation between TOF and the single-atom dominance index (I_{ratio}) for the Ir_{SA+AC}/NC catalyst series. (b) Correlation between TOF and the coexistence intensity (I_{prod}) for the same catalyst series. (c) APRM activity of separately tested Ir_{SA}/NC and Ir_{AC}/NC, as well as their physical mixtures with varying amounts of inert NC. (d) Corresponding APRM selectivity of Ir_{SA}/NC, Ir_{AC}/NC, and their NC-diluted mixtures.

Supplementary Note 1

We examined correlations between TOF and coordination-number (CN)–derived descriptors and introduced two interfacial metrics. The single-atom dominance index, I_{ratio} ($I_{ratio} = \frac{CN_{Ir-N}}{CN_{Ir-Ir}}$), quantifies enrichment of single atoms relative to clusters, I_{prod} ($I_{prod} = CN_{Ir-N} \times CN_{Ir-Ir}$), gauges SA-AC co-presence (a necessary condition for synergy). Activity increases primarily with the enrichment of single atoms relative to particles (Fig. S10a), whereas simply increasing SA-AC co-presence affords no net kinetic benefit (Fig. S10b). Consistent with the need for interfacial coupling, physically separating the two sites (Ir_{SA}/NC and Ir_{AC}/NC) lowers the H_2 production rate from 346.9 to 153 mol H_2 mol Ir^{-1} h^{-1} relative to the synergistic Ir_{SA+AC}/NC catalyst, demonstrating that proximity between Ir SA and Ir AC is essential for high APRM activity and selectivity (Figs. S10c and d).

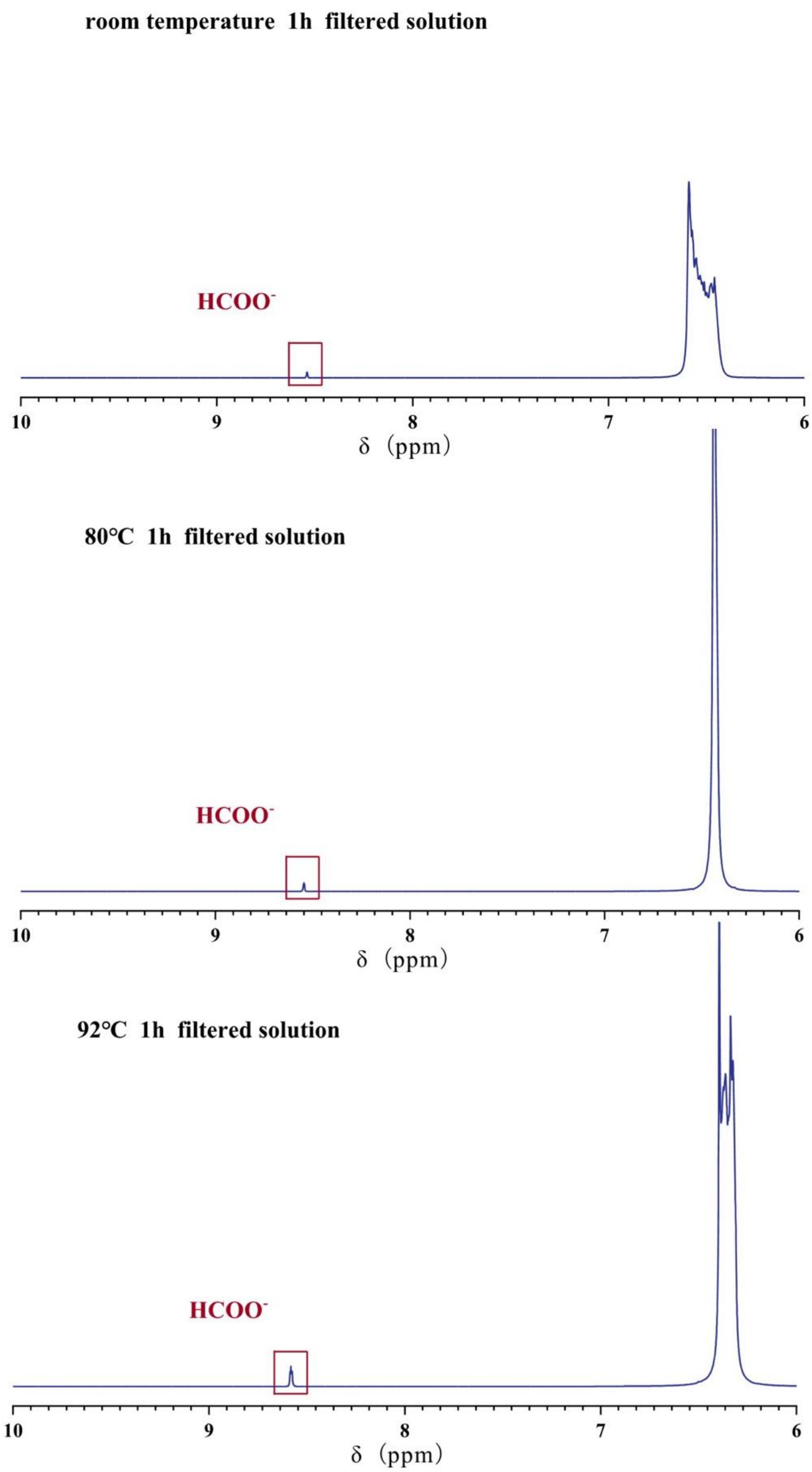

Figure S11. ^{1}H NMR spectra (MeOH/H_2O 4:1, 8 M KOH, CD_3OD) of Ir_{SA+AC}/NC reaction mixture. Spectra were recorded at room temperature; the values reported on the spectra refer to the reaction temperature at the time of sample withdrawal.

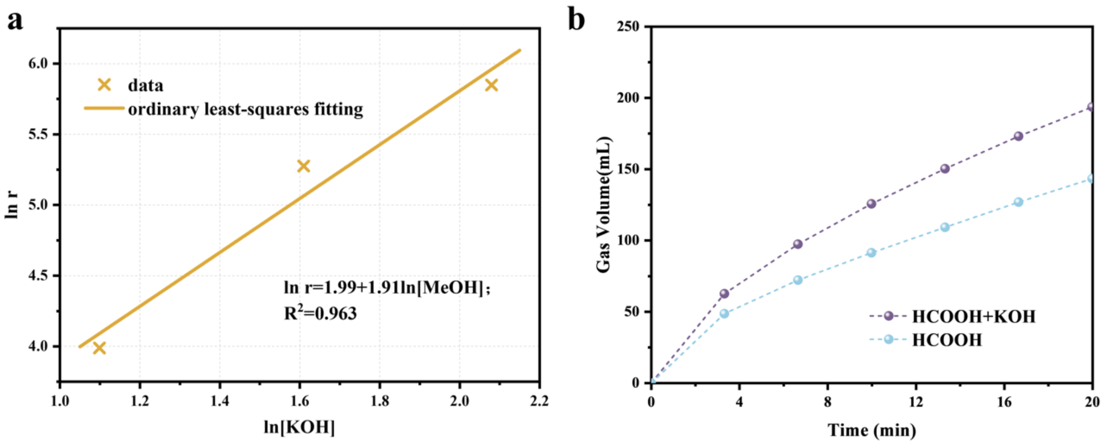

Figure S12. (a) Dependence of the APRM rate on KOH concentration over Ir_{SA+AC}/NC; slopes of the fitted lines give the reaction orders. (b) FAD activity of Ir_{SA+AC}/NC with and without base.

Supplementary Note 2

KOH exerts a pronounced kinetic influence. The rate shows an apparent near-second-order dependence on alkalinity (n=1.9) and is therefore highly sensitive to KOH (Fig. S12a), within the examined range, doubling [KOH] affords a ~3-4-fold rate increase. The accelerating effect of KOH is attributed to two cooperative contributions:

(1) Solvation/salting-out and vapor–liquid equilibrium modulation. KOH strongly hydrates water and salts out methanol, diminishing solvent activities and shifting the vapor liquid equilibrium to higher temperatures. Consequently, the mixture attains a total pressure of 1 atm only at elevated temperatures (*J. Solution Chem.* 2010, 39, 335–342; *J. Chem. Eng. Data* 1979, 24 (1), 9–11; *J. Chem. Eng. Data* 2003, 48, 344–346), and the measured liquid-phase temperature can exceed 90 ℃ under ambient pressure (Table S4), providing a higher effective reaction temperature without external pressurization.

(2) Base-promoted speciation and acceleration of the key elementary step. Under alkaline conditions, alcohols are rapidly deprotonated to alkoxides (CH_3O^-), and formic acid is quantitatively converted to formate ($HCOO^-$). In formic-acid dehydrogenation catalysis (including the present system), dehydrogenation of formate is a key elementary step; strong base shifts the equilibrium from molecular HCOOH to the more reactive $HCOO^-$ intermediate enhancing overall hydrogen-evolution rates (*Chem. Soc. Rev.* 2016, 45, 3954–3988; *Chem. Rev.* 2018, 373, 317–332; *Chimia* 2011, 65, 214–218; *Nat. Commun.* 2016, 7, 11308). Consistent with our experiment, addition of KOH uniformly accelerates the overall process, with a particularly pronounced effect on the putative rate-determining formate-dehydrogenation step (Fig. S12b).

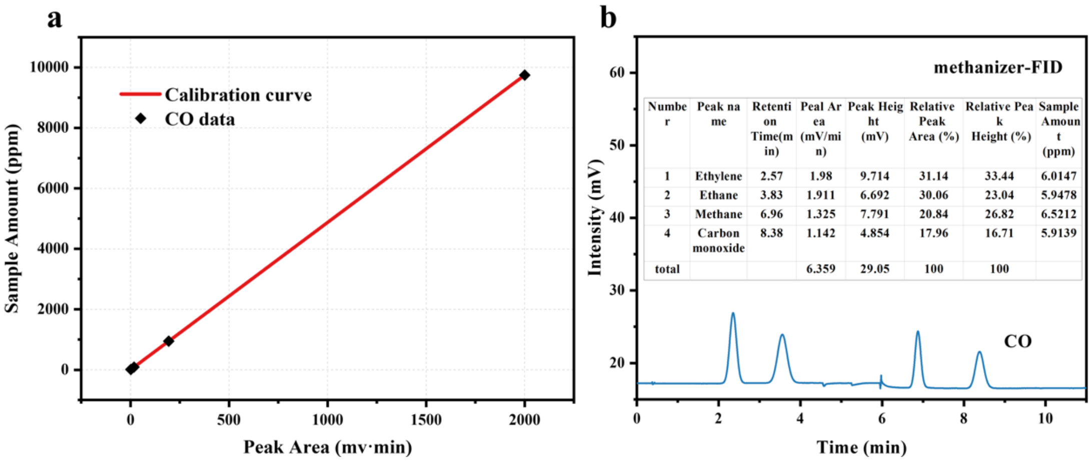

Number	Peak name	Retention Time(min)	Peal Area (mV/min)	Peak Height (mV)	Relative Peak Area (%)	Relative Peak Height (%)	Sample Amount (ppm)
1	Ethylene	2.57	1.98	9.714	31.14	33.44	6.0147
2	Ethane	3.83	1.911	6.692	30.06	23.04	5.9478
3	Methane	6.96	1.325	7.791	20.84	26.82	6.5212
4	Carbon monoxide	8.38	1.142	4.854	17.96	16.71	5.9139
total			6.359	29.05	100	100	

Figure S13. (a) Calibration curves for CO on the GC using different certified standard gases (10, 100, 1,000, and 10,000 ppm). (b) GC response for low concentration CO.

Supplementary Note 3

"CO-free" denotes that CO in the product gas is below the detection limit of the gas chromatograph (GC) equipped with a methanizer.

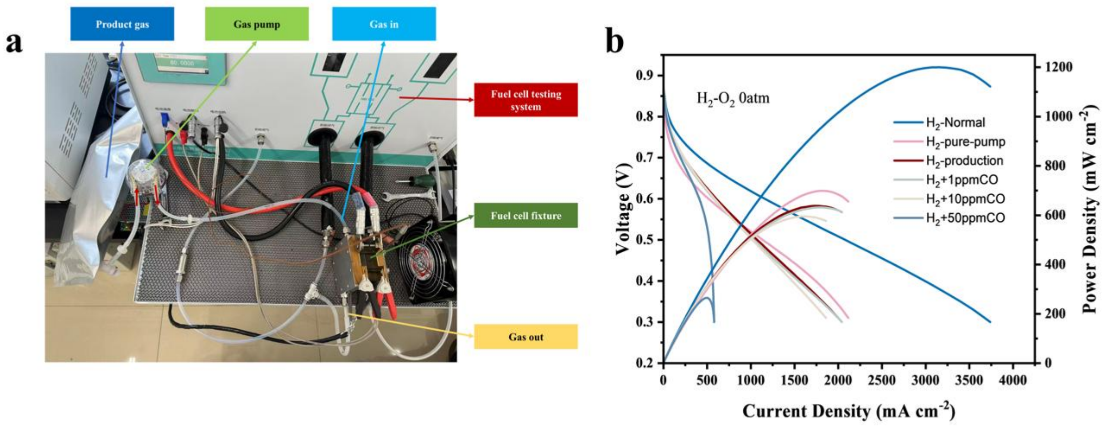

Figure S14. (a) Photograph of the fuel cell testing system employing a pump-fed product-gas supply to the anode. (b) Discharge polarization and corresponding power-density curves of Pt/C operated under different gas feeds.

Supplementary Note 4

Because the collected product gas could not meet the ≥0.35 MPa inlet requirement, it was supplied to the PEMFC anode by a pump without humidification (Fig. S14a), that inherently underperforms the pressurized, humidified benchmark. The humidified H_2 benchmark reached 1200 mW cm^{-2}; switching to dry, pump-fed H_2 incurred a ~42% loss (698.9 mW cm^{-2}), consistent with membrane dehydration and lower anode partial pressure. Under the same delivery, the product gas imposed only a modest penalty (638.2 mW cm^{-2}) relative to pump-fed H_2. Systematic CO doping under dry, pump-fed delivery showed small penalties at 1–10 ppm (632.2 and 593.2 mW cm^{-2}) but a severe loss at 50 ppm (264.95 mW cm^{-2}), evidencing tolerance at low-ppm levels with rapid inhibition at higher CO (Fig. S14b). Overall, PEMFC operation on the produced gas (no cleanup) is feasible, performance is limited primarily by delivery (humidity/pressure), not impurity content.

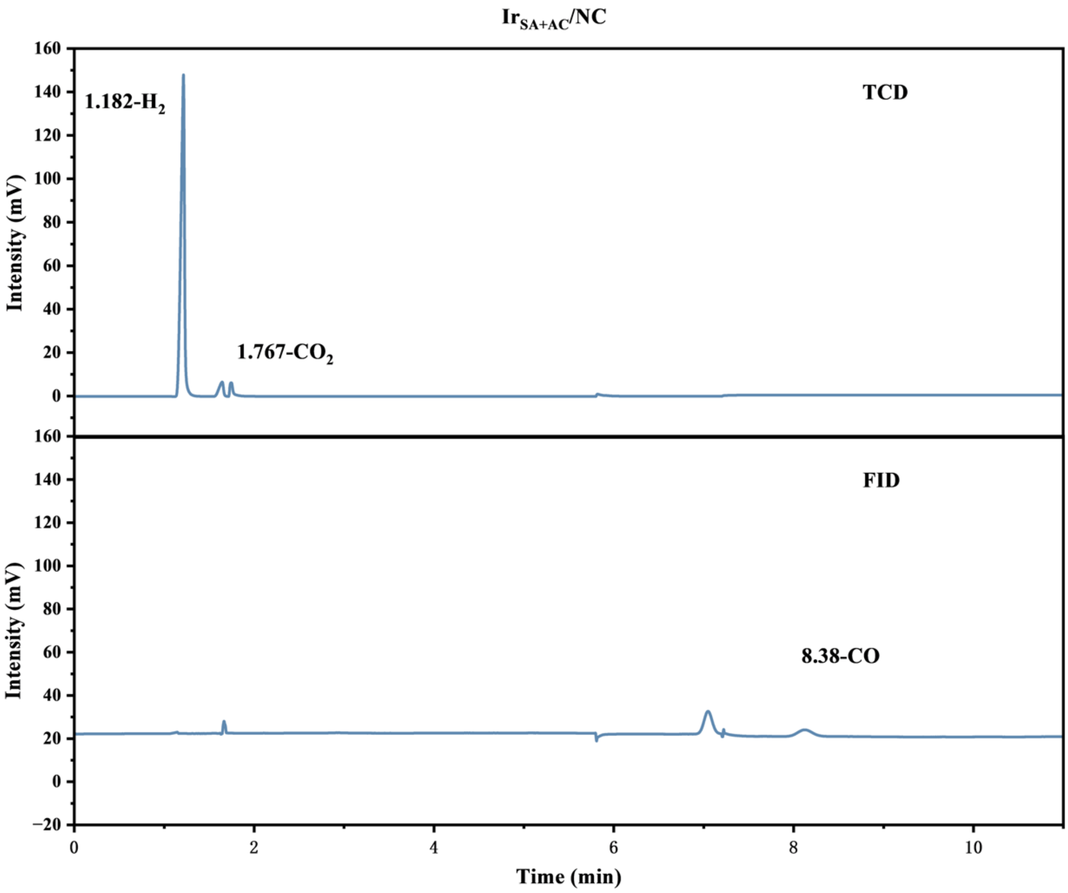

Number	Peak Name	Retention Time (min)	Peak Area (mV·min)	Peak Height (mV)	Relative Peak Area (%)	Relative Peak Height (%)	Sample Amount (ppm)
1	hydrogen	1.182	6.951	148.046	95.86	95.92	56414.3811
2	carbon dioxide	1.767	0.300	6.294	4.14	4.08	917.3205
3	carbon monoxide	8.38	-	-	-	-	-
total			7.252	154.340	100	100	

Figure S15. Reaction producing primarily H_2, with CO not detected.

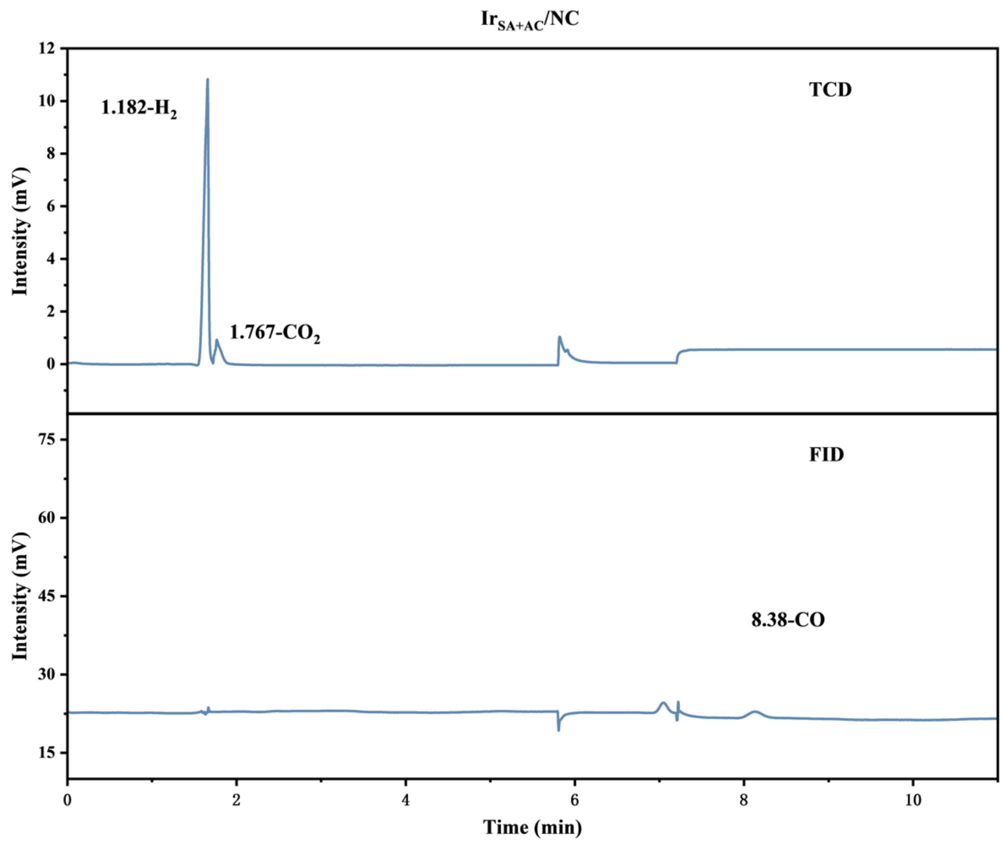

Number	Peak Name	Retention Time (min)	Peak Area (mV·min)	Peak Height (mV)	Relative Peak Area (%)	Relative Peak Height (%)	Sample Amount (ppm)
1	hydrogen	1.182	0.001	0.019	1.03	2.12	5.7205
2	carbon dioxide	1.767	0.069	0.897	98.97	97.88	5697.0236
3	carbon monoxide	8.38	-	-	-	-	-
total			0.069	0.917	100	100	

Figure S16. The gas released from the reaction solution after the reaction is primarily CO_2. In an alkaline environment, most of the carbon dioxide is absorbed. After the reaction, the CO_2 in the solution is released by adding dilute acid.

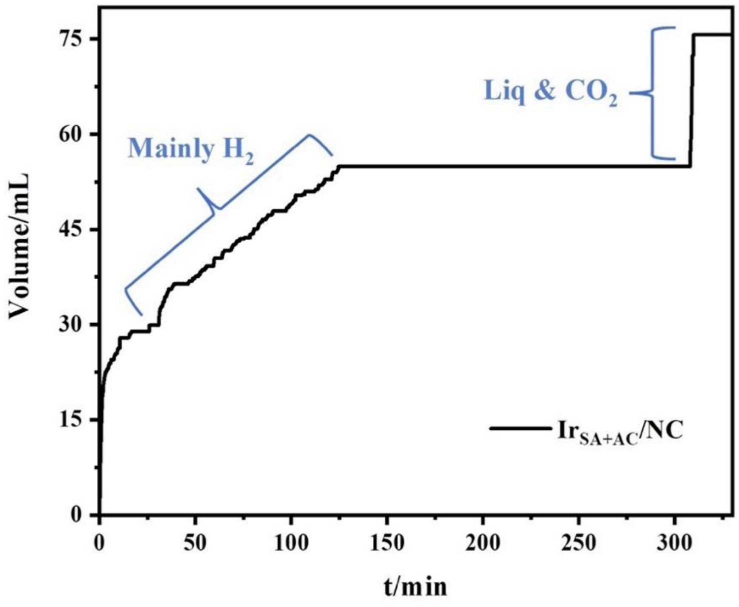

Figure S17. The reaction produces a 2.87:1 ratio of H_2/CO_2. After the reaction stops, the system is naturally cooled to room temperature, and dilute acid is added to the solution to release CO_2, which is present in the form of carbonate.

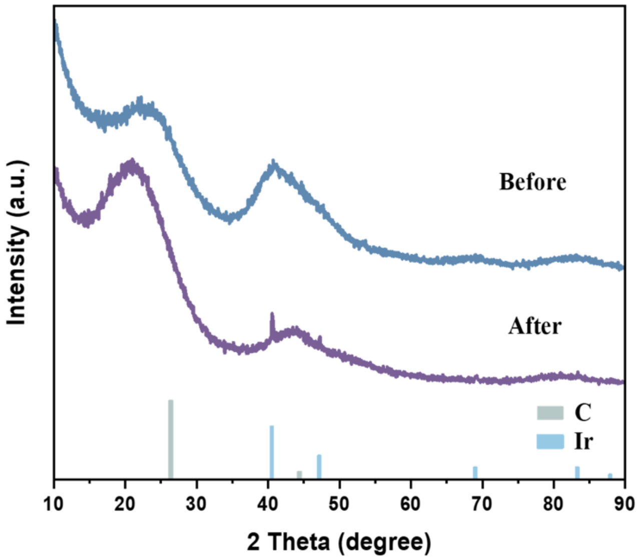

Figure S18. XRD patterns of Ir_{SA+AC}/NC before and after cyclic stability testing.

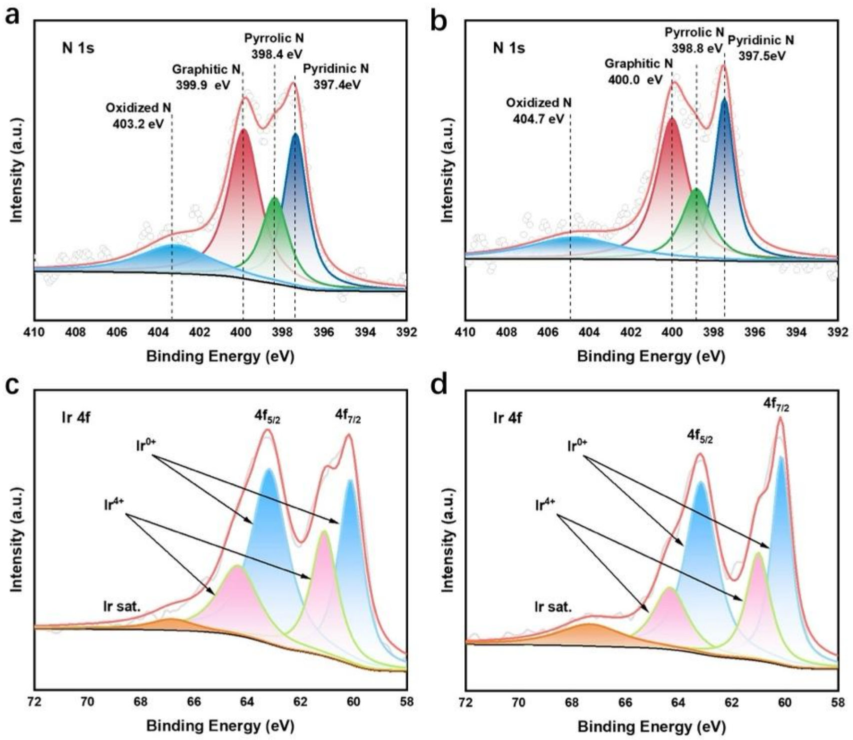

Figure S19. (a, b) N 1s XPS spectra for Ir_{SA+AC}/NC before and after cyclic stability testing. (c, d) Ir 4f XPS spectra for Ir_{SA+AC}/NC before and after cyclic stability testing.

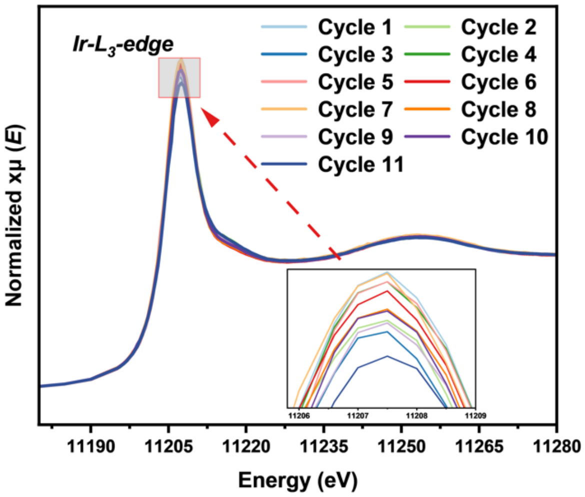

Figure S20. Normalized Ir L_3-edge XANES spectra of Ir_{SA+AC}/NC after different reaction cycles.

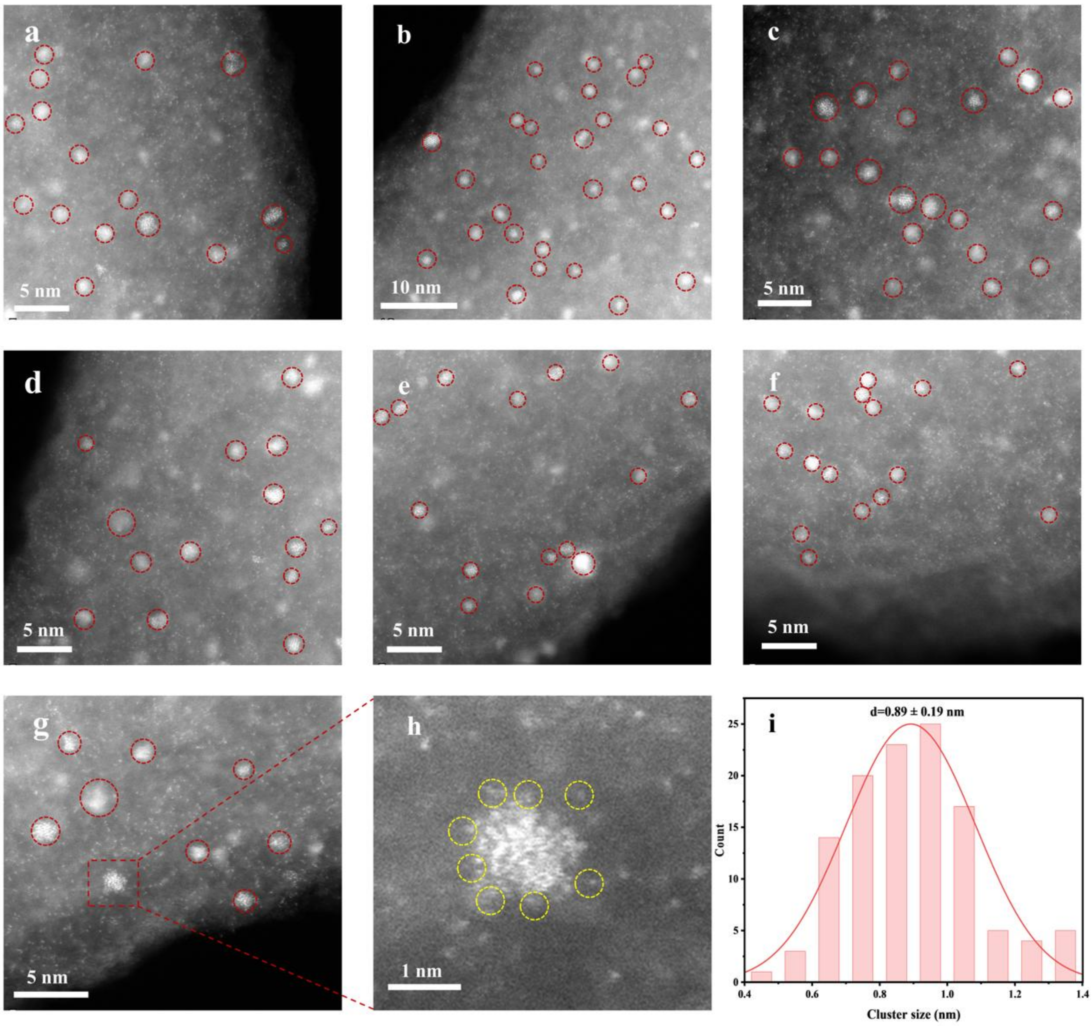

Figure S21. (a-h) High-resolution HAADF–STEM images of Ir_{SA+AC}/NC after 11 cycle operation, taken from randomly selected regions, verify the coexistence of single atoms and clusters. (i) The average Ir cluster diameter is ~0.89 nm, based on statistics from the red-circled regions.

Supplementary Note 5

ICP of the post-reaction liquors shows ~0.4% Ir loss per cycle and ~4.8% in total; thus, Ir leaching alone cannot account for the observed ~17% decrease in TOF when rates are normalized to the initial Ir loading (Table S6). XAFS collected after each cycle shows a gradual decrease in white-line intensity with a concomitant increase in the Ir–Ir coordination number, consistent with partial evolution from single atoms to atomically dispersed clusters (Fig. S20, Table S7). HAADF-STEM after 11 cycles reveals growth of the characteristic feature size from 0.58 nm (fresh) to 0.89 nm (spent), remaining within the sub-2-nm regime and without emergence of large nanoparticles (Fig. S21). This controlled SA to AC restructuring rather than extensive Ir dissolution—rationalizes the moderate activity decay while preserving 100% H_2 selectivity.

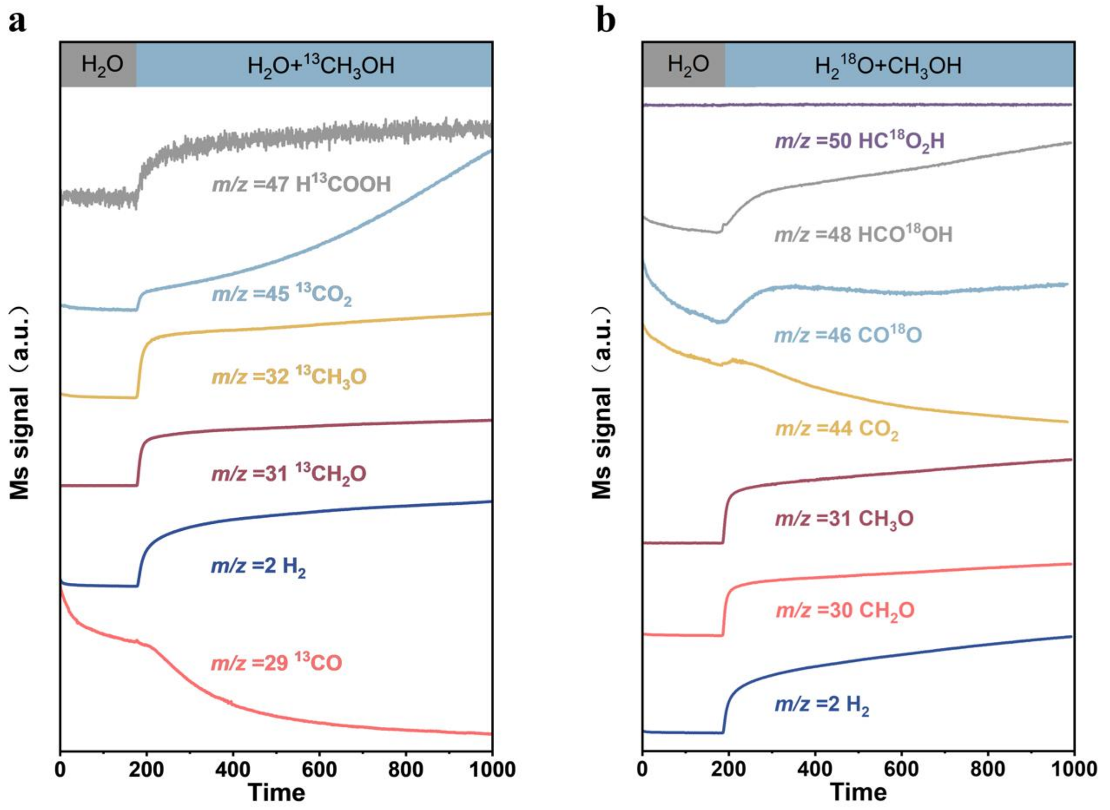

Figure S22. (a) In situ mass spectrometry of APRM over Ir_{SA+AC}/NC using $^{13}CH_3OH$ as the isotopic tracer. (b) In situ mass spectrometry of APRM over Ir_{SA+AC}/NC using $H_2{}^{18}O$ as the isotopic tracer.

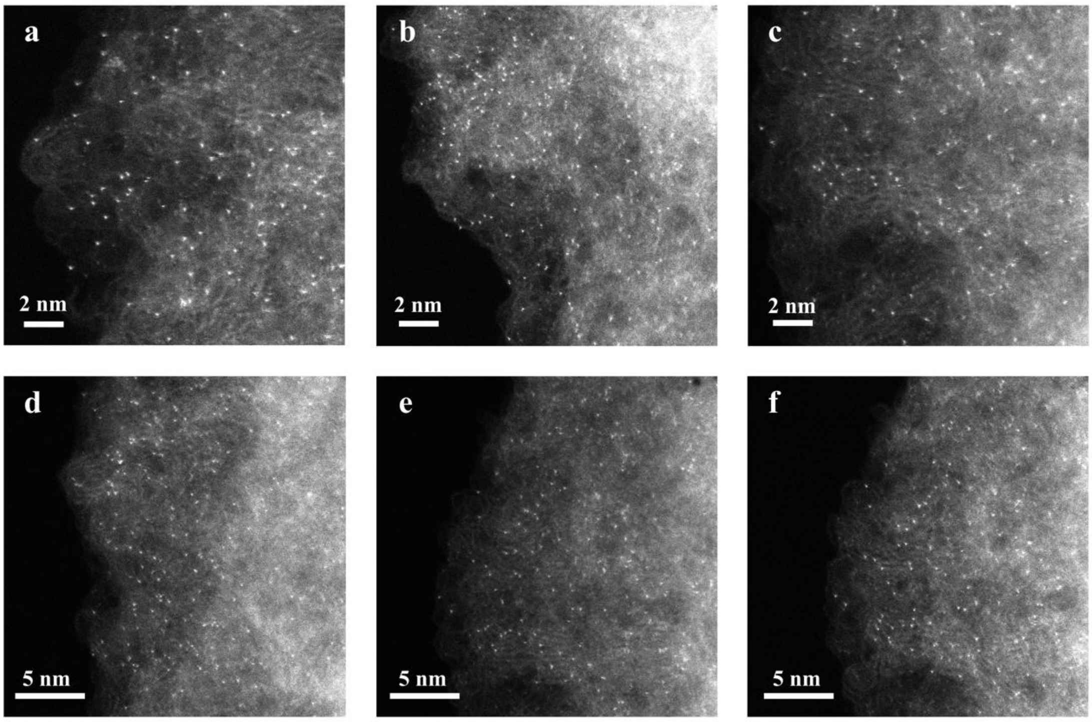

Figure S23. High-resolution HAADF-STEM images of Ir_{SA}/NC from randomly selected regions verified Ir species were atomically dispersed in the substrate and no Ir nanoparticles were formed.

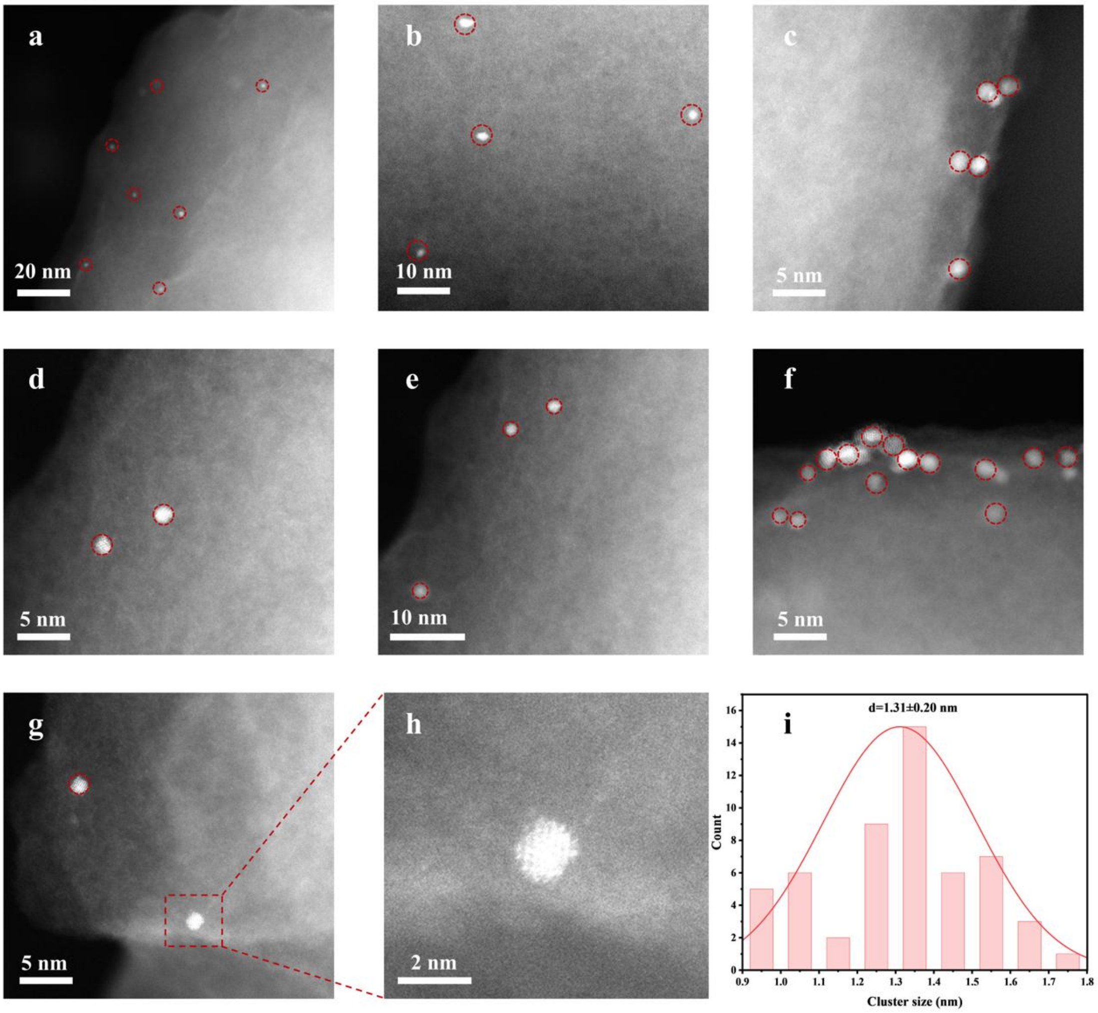

Figure S24. (a-h) High-resolution HAADF–STEM images of Ir_{AC}/NC from randomly selected regions verified the exclusive presence of Ir nanoparticles without any detectable single-atom species. (i) The average Ir cluster diameter is ~1.31 nm, based on statistics from the red-circled regions.

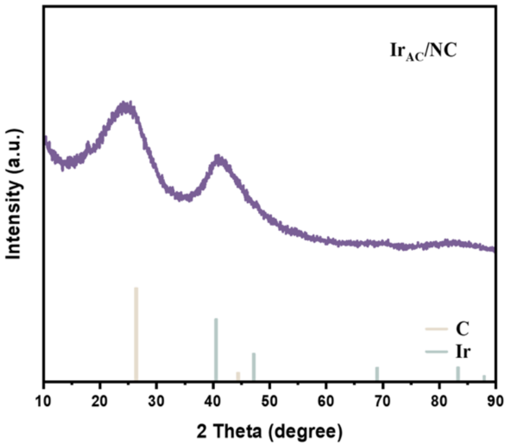

Figure S25. X-ray diffraction (XRD) patterns of Ir_{AC}/NC.

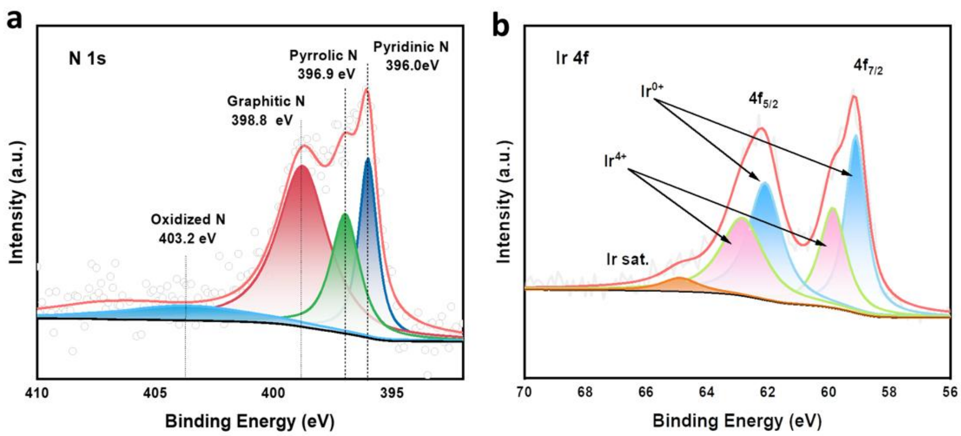

Figure S26. High-resolution XPS spectra of Ir_{AC}/NC: (a) N 1s and (b) Ir 4f regions.

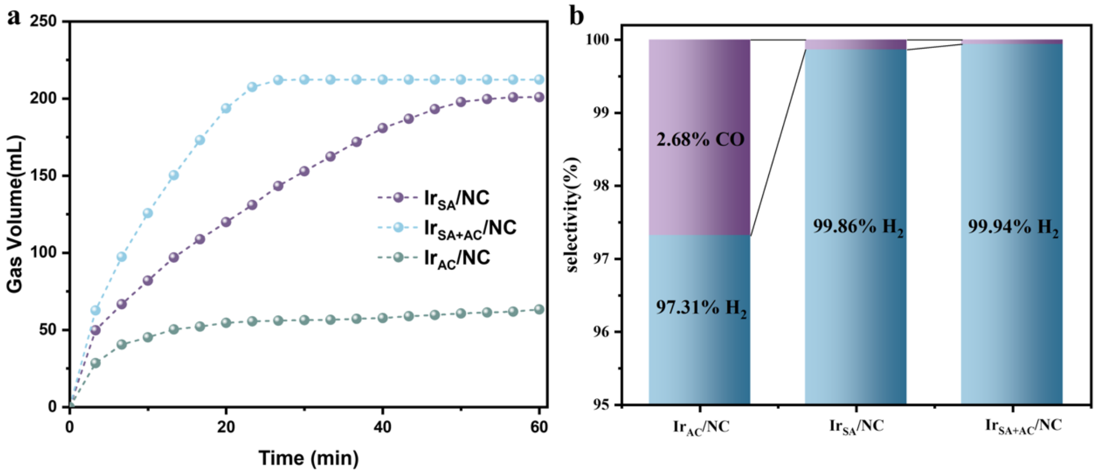

Figure S27. (a) Formic acid decomposition (FAD) activity of Ir_{SA}/NC, Ir_{AC}/NC, and Ir_{SA+AC}/NC. (b) Corresponding FAD selectivity for Ir_{SA}/NC, Ir_{AC}/NC, and Ir_{SA+AC}/NC.

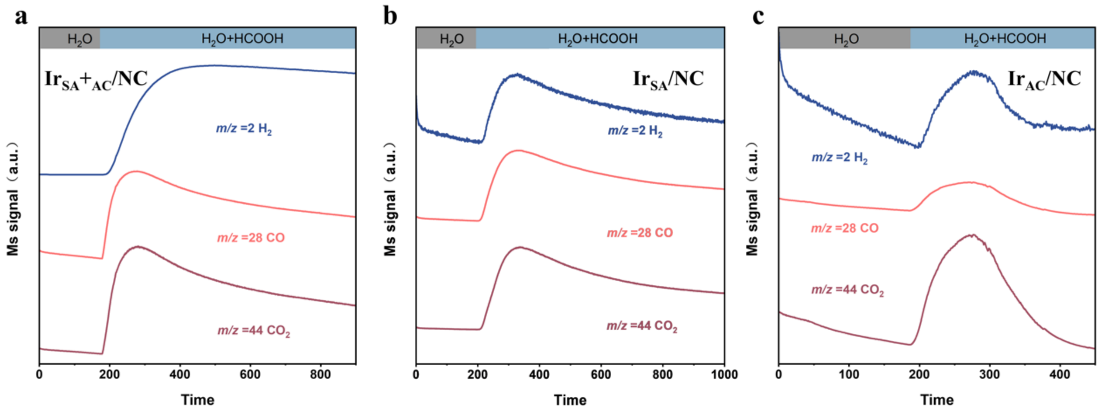

Figure S28. (a–c) In situ mass spectrometry of FAD over Ir_{SA+AC}/NC, Ir_{SA}/NC, and Ir_{AC}/NC.

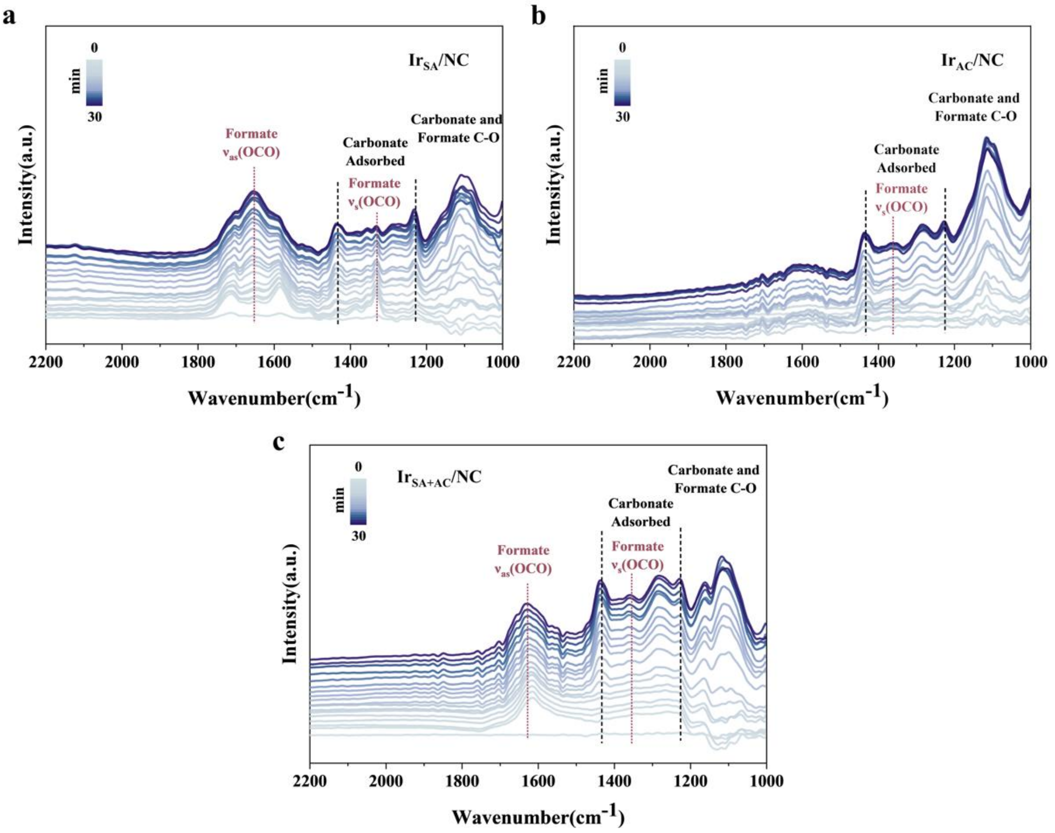

Figure S29. (a-c) Time-resolved in situ ATR-SEIRAS spectra of Ir_{SA}/NC, Ir_{AC}/NC, and Ir_{SA+AC}/NC collected during FAD at room temperature.

Supplementary Note 6

For formic acid decomposition (FAD) site identity governs the dehydrogenation–dehydration balance and CO tolerance. Under FAD, Ir_{SA+AC}/NC delivers the highest rate and reaches 86.3% conversion; Ir_{SA}/NC is somewhat slower but attains a similar final conversion, whereas Ir_{AC}/NC deactivates rapidly and plateaus near 24.3% (Fig. S27a). Online GC reveals an elevated CO fraction on Ir_{AC}/NC (Fig. S27b), implicating greater dehydration (HCOOH → CO + H_2O) and CO-induced poisoning of cluster sites.

In situ MS shows sustained m/z = 2 (H_2) and 44 (CO_2) on Ir_{SA+AC}/NC and Ir_{SA}/NC—with higher intensities on Ir_{SA+AC}/NC—indicative of faster dehydrogenation (Figs. S28a, b). By contrast, Ir_{AC}/NC exhibits only transient m/z = 2, 44 that rapidly decay (Fig. S28c), consistent with inefficient FAD and fast deactivation, plausibly via CO poisoning; a weak yet reproducible m/z = 28 (CO) appears on all three but, given the high sensitivity of MS to short-lived/low-abundance species, does not contradict dehydrogenation dominance on Ir SA-containing samples.

Complementary in situ ATR-SEIRAS in aqueous HCOOH reveals carbonate/bicarbonate features ($\nu_3(CO_3^{2-})$ ≈ 1430-1440 cm^{-1}; HCO_3^- ≈ 1228–1232 cm^{-1}) and a band near ≈1108 cm^{-1} (formate C–O), evidencing formic-acid dehydrogenation to CO_2 on all catalysts (Figs. S29a-c). Crucially, the coordination motif differentiates the sites: Ir_{AC}/NC is dominated by bidentate formate (ν_s(OCO) ≈

1355 cm^{-1}; monodentate ν_{as}(OCO) ≈ 1641 cm^{-1} nearly absent), whereas Ir_{SA}/NC exhibits a strong ν_{as}(OCO) ≈ 1641 cm^{-1} and ν_{s}(OCO) ≈ 1330 cm^{-1} assignable to linearly bound formate. Ir_{SA+AC}/NC presents a superposition of both signatures, consistent with tandem of adjacent Ir SA and Ir AC. On the basis of these spectroscopic assignments, we infer that monodentate formate—prevailing on Ir SA sites and associated with faster dehydrogenation to CO_2—leads to higher CO tolerance and accelerated HCOOH dehydrogenation, whereas the predominance of bidentate formate on Ir AC sites, which is more closely linked to CO-forming pathways, renders these sites more vulnerable to CO-induced deactivation.

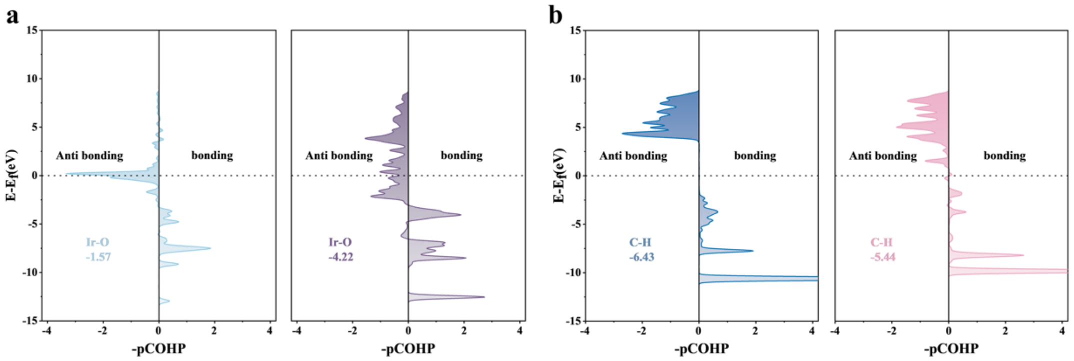

Figure S30. (a) –COHP of the Ir–O bond for CH_3O^* adsorption on Ir SA (left) and Ir AC (right) sites of Ir_{SA+AC}/NC. (b) –COHP of the C–H bond for HCOO* adsorption on Ir AC (left) and Ir SA (right) sites of Ir_{SA+AC}/NC.

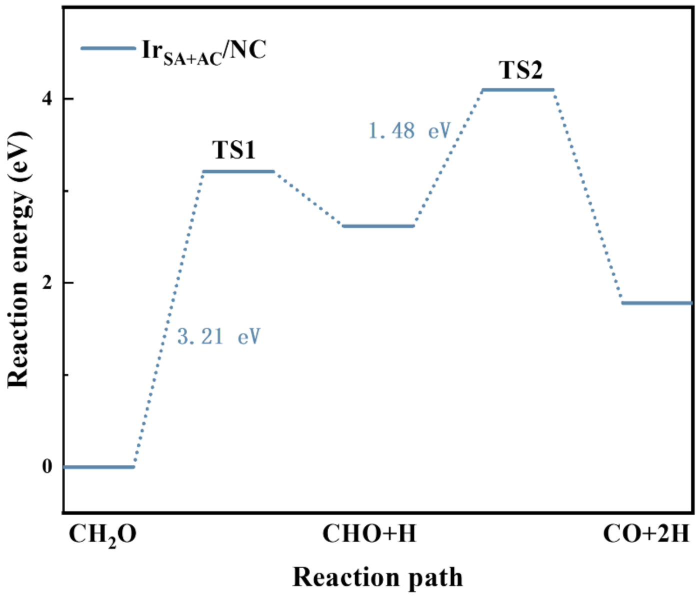

Figure S31. Energy profiles for the dissociation of CH_2O into CO and H atoms on Ir_{SA+AC}/NC.

Table S1. Ir contents in catalysts determined by ICP-MS

Samples	Elements	Content (wt. %)
0.5wt% Ir_{SA}/NC	Ir	0.31
1wt% Ir_{SA}/NC	Ir	0.71
2wt% Ir_{SA}/NC	Ir	1.28
3wt% Ir_{SA+AC}/NC	Ir	2.31
4wt% Ir_{SA+AC}/NC	Ir	3.34
5wt% Ir_{SA+AC}/NC	Ir	3.91
7wt% Ir_{SA+NP}/NC	Ir	5.28
8wt% Ir_{SA+NP}/NC	Ir	6.05
9wt% Ir_{SA+NP}/NC	Ir	6.78
Ir_{AC}/NC	Ir	3.58

Supplementary Note 7

Unless otherwise specified, Ir_{SA}/NC refers to 0.5wt% Ir_{SA}/NC, Ir_{SA+AC}/NC refers to 4wt% Ir_{SA+AC}/NC, and Ir_{SA+NP}/NC refers to 8wt% Ir_{SA+NP}/NC. These designations are used consistently throughout the main text and Supporting Information.

Table S2. EXAFS fitting parameters at the Ir L_3-edge of Ir_{SA}/NC, Ir_{SA+AC}/NC and Ir_{SA+NP}/NC.

samples	Scattering path	CN	ΔR	R (Å)	σ^2 (10^{-3}Å^2)	R-factor
Ir_{SA}/NC	Ir-N	4.9	0.006	2.01	6.5	0.015
4wt% Ir_{SA+AC}/NC	Ir-N	4.2	-0.001	2.01	6.3	0.01615
	Ir-Ir	2.5	-0.039	2.70	2.9	
5wt% Ir_{SA+AC}/NC	Ir-N	3.2	-0.012	1.99	3.0	0.00959
	Ir-Ir	4.2	-0.114	2.61	9.0	
6wt% Ir_{SA+AC}/NC	Ir-N	3.2	-0.007	2.00	3.0	0.01235
	Ir-Ir	5.3	-0.128	2.59	12.7	
7wt% Ir_{SA+NP}/NC	Ir-N	3.1	-0.011	2.00	3.0	0.00851
	Ir-Ir	7.5	-0.172	2.55	16.7	
8wt% Ir_{SA+NP}/NC	Ir-N	1.9	-0.09	2.00	9.8	0.0066
	Ir-Ir	8.0	-0.03	2.71	3.5	

Table S3. Various catalysts for generating hydrogen through APRM.

Entry	Catalyst	*additive*	*Base*	*T (inner, C °)*	*TOF (mol H_2 per mol Ir per hour)*
1	Ir_{SA+AC}/NC	-	8M KOH	95	346.9
2	Ir_{SA+AC}/NC	-	5M KOH	91.1	195.6
3	Ir_{SA+AC}/NC	-	3M KOH	75.1	54
4	Ir_{SA+AC}/NC	-	-	70	-
5[a]	Ir_{SA+AC}/NC	-	8M KOH	91.2	485.4
6[b]	Ir_{SA+AC}/NC	-	8M KOH	95.3	271.3
7[c]	Ir_{SA+AC}/NC	-	8M KOH	94.1	260.2
8[d]	Ir_{SA+AC}/NC	-	8M KOH	95	225.9
9[e]	Ir_{SA+AC}/NC	-	8M KOH	95	-
10[f]	NC	-	8M KOH	95	-
11	Ir_{SA+AC}/NC	5ml Triglyme	5M KOH	100	76.3
12	Ir_{SA+AC}/NC	5ml Triglyme	3M KOH	98	45.8
13	Ir_{SA+AC}/NC	5ml Triglyme	1M KOH	87.1	4.4
14	Ir_{SA+AC}/NC	20ml Triglyme	1M KOH	90.5	32.6
15	Ir_{SA+AC}/NC	20ml Triglyme	0.3M KOH	85.9	-
16	0.5wt% Ir_{AC}/NC	-	8M KOH	95	-
17	3wt% Ir_{AC}/NC	-	8M KOH	95	-
18	6wt% Ir_{AC}/NC	-	8M KOH	95	-

Reaction conditions: n(MeOH):(H_2O) = 4:1 (except for entries 5-9), 5 mL total volume of MeOH /H_2O, 20 mg catalyst, T set = 100 ℃; reaction for 2 h.

[a-e]The reactant MeOH:H_2O molar ratios = 9:1, 7:3, 6:4, 1:1, and 1:4, respectively.

[f]Pure NC without Ir content.

Supplementary Note 8

In a standard reaction vessel, equipped with a thermometer, a total of 5 mL of the specified MeOH/H_2O ratio and the stated amount of catalyst were introduced. The vessel was heated to the temperature set in the thermostat. The inner temperature was recorded after 30 min of stirring at the set temperature. Under certain conditions, increasing the base concentration enhances the catalyst's activity, and as the base concentration increases, the inner temperature (T inner) also rises.

Table S4. Solution temperatures at different MeOH/H_2O ratios, base concentrations, and Triglyme concentrations.

Entry	MeOH/H_2O ratio	*Base*	*additive*	*T (inner, C °)*
1	9:1	-	-	69.5
2	9:1	3M NaOH	-	74.5
3	9:1	5M KOH	-	85
4	9:1	7M KOH	-	88
5	9:1	8M KOH	-	91.2
6	4:1	-	-	70
7	4:1	5M NaOH	-	78.5
8	1:1	-	-	77.5
9	1:1	7M KOH	-	87.2
10	1:1	7M KOH	5ml Triglyme	90.6

Table S5. Comparison of H_2 Production Activity and Reaction Conditions (≤100 °C) for Various Hydrogen-Production Routes

Sample	Reaction conditions	Hydrogen production route	H_2 rate (μmol gcat^{-1} h^{-1})	Reference
Ir_{SA+AC}/NC	95 ℃; ambient pressure	Thermal	60,300	This Work
Cu-WC/W	25 ℃; ambient pressure	Plasmonic photocatalysis	2,176.70	*Proc. Natl. Acad. Sci. U.S.A.* 2023, 120, e2212075120
Cu/TiO_2 (PC50)	30 ℃; ambient pressure	Photocatalysis	25,487	*Green Chem.* 2022, 24, 8345–8354
MgO nanocrystals	25 ℃; ambient pressure	Photocatalysis	320	*Sci. Adv.* 2016, 2, e1501425
Pt/H_2-In_2O_3	~100 ℃; ambient pressure	Thermal	206	*J. Alloys Compd.* 2025, 1011, 178458
Au/TiO_2	25 ℃; ambient pressure	Photocatalysis	1,866	*J. Hazard. Mater.* 2013, 263, 2–10
PtCu-TiO_2	70 ℃; ambient pressure	Photocatalysis	2,383.90	*Nat. Mater.* 2023, 22, 619–626
MetCN30	35 ℃; ambient pressure	Photocatalysis	35,600	*Angew. Chem. Int. Ed.* 2025, 64, e202418677

Supplementary Note 9

To benchmark our catalyst, we surveyed reports of heterogeneous methanol–water reforming under purely thermal conditions with inclusion criteria of T ≤100 °C (or the lowest reported) and found no prior examples achieving efficient reforming below 100 ℃. Notably, even the rare ~100 ℃ thermal case—Pt nanoparticles on defect-engineered In_2O_3—delivers productivities orders of magnitude lower than in this work. For completeness, we also compare selected multi-field systems (e.g., photothermal).

Table S6. Correlation among metal leaching, catalyst structure, and catalytic activity of spent Ir_{SA+AC}/NC catalysts.

samples	Loss this cycle (%)	Ir Content (wt%)	TOF (h-)	CN_{Ir-N}	CN_{Ir-Ir}
Cycle 1	0.351	3.3283	270.2	3.61395	4.39163
Cycle 2	0.23	3.3207	234.5	3.41131	4.84574
Cycle 3	0.341	3.3093	223.65	3.53896	4.40402
Cycle 4	0.042	3.3079	239.85	3.60282	4.56174
Cycle 5	1.042	3.2734	207.05	3.58021	4.79825
Cycle 6	0.514	3.2566	232.95	3.58432	4.13984
Cycle 7	0.353	3.2451	260.9	3.54867	4.1474
Cycle 8	0.421	3.2314	287.8	3.42416	4.36412
Cycle 9	0.085	3.2286	246.2	3.29019	3.28814
Cycle 10	0.629	3.2083	253.6	3.27652	3.82031
Cycle 11	0.482	3.1928	225	3.48758	3.90207

Table S7. Structural parameters of spent Ir_{SA+AC}/NC catalysts obtained from EXAFS fitting.

samples	Scattering path	*CN*	*ΔR*	*R (Å)*	σ^2 (10^{-3}Å^2)	*R-factor*
Cycle 1	Ir-N	3.61395	-0.02385	1.98785	3.06	0.0182219
	Ir-Ir	4.39163	-0.43099	2.29411	15.9	
Cycle 2	Ir-N	3.41131	-0.02938	1.98232	3.05	0.0139036
	Ir-Ir	4.84574	-0.43309	2.29201	15.41	
Cycle 3	Ir-N	3.53896	-0.02852	1.98318	3.05	0.0158566
	Ir-Ir	4.40402	-0.4286	2.2965	14.56	
Cycle 4	Ir-N	3.60282	-0.01118	2.00052	4.74	0.0123123
	Ir-Ir	4.56174	-0.45906	2.26604	18.48	
Cycle 5	Ir-N	3.58021	-0.01204	1.99966	4.65	0.0121141
	Ir-Ir	4.79825	-0.4631	2.262	18.2	
Cycle 6	Ir-N	3.58432	-0.01	2.0017	4.63	0.0117834
	Ir-Ir	4.13984	-0.44598	2.27912	16.52	
Cycle 7	Ir-N	3.54867	-0.00717	2.00453	4.22	0.0180807
	Ir-Ir	4.1474	-0.45	2.2751	19.01	
Cycle 8	Ir-N	3.42416	-0.01485	1.99685	3.22	0.0178259
	Ir-Ir	4.36412	-0.43737	2.28773	17.39	
Cycle 9	Ir-N	3.29019	-0.01198	1.99972	3.96	0.0149797
	Ir-Ir	3.28814	-0.45502	2.27008	14.44	
Cycle 10	Ir-N	3.27652	-0.01163	2.00007	3.44	0.0150675
	Ir-Ir	3.82031	-0.44492	2.28018	15.51	
Cycle 11	Ir-N	3.48758	-0.01847	1.99323	4.52	0.0144118
	Ir-Ir	3.90207	-0.44094	2.28415	14.9	
After 65h	Ir-N	2.7114	-0.01397	1.99773	8.25	0.0120701

Ir-Ir	9.33582	-0.11141	2.61369	13.08

References

1. Segall MD, Philip JDL, Probert MJ *et al.* First-principles simulation: ideas, illustrations and the CASTEP code. *J Phys: Condens Matter*. 2002; **14**(11): 2717.
2. Ahmed Adllan A, Dal Corso A. Ultrasoft pseudopotentials and projector augmented-wave data sets: application to diatomic molecules. *J Phys: Condens Matter*. 2011; **23**(42): 425501.
3. Perdew JP, Burke K, Ernzerhof M. Generalized Gradient Approximation Made Simple. *Phys Rev Lett*. 1996; **77**(18): 3865-3868.
4. Tkatchenko A, Scheffler M. Accurate Molecular Van Der Waals Interactions from Ground-State Electron Density and Free-Atom Reference Data. *Phys Rev Lett*. 2009; **102**(7): 073005.
5. Govind N, Petersen M, Fitzgerald G *et al.* A generalized synchronous transit method for transition state location. *Computational Materials Science*. 2003; **28**(2): 250-258.
